# Towards Systematic Discovery of Signaling Networks in Budding Yeast Filamentous Growth Stress Response Using Interventional Phosphorylation Data

**DOI:** 10.1371/journal.pcbi.1003077

**Published:** 2013-06-27

**Authors:** Yan Zhang, Hye Kyong Kweon, Christian Shively, Anuj Kumar, Philip C. Andrews

**Affiliations:** 1 Department of Computational Medicine and Bioinformatics, University of Michigan Medical School, Ann Arbor, Michigan, United States of America; 2 Department of Biological Chemistry, University of Michigan Medical School, Ann Arbor, Michigan, United States of America; 3 Department of Molecular, Cellular, and Developmental Biology, University of Michigan, Ann Arbor, Michigan, United States of America; University College London, United Kingdom

## Abstract

Reversible phosphorylation is one of the major mechanisms of signal transduction, and signaling networks are critical regulators of cell growth and development. However, few of these networks have been delineated completely. Towards this end, quantitative phosphoproteomics is emerging as a useful tool enabling large-scale determination of relative phosphorylation levels. However, phosphoproteomics differs from classical proteomics by a more extensive sampling limitation due to the limited number of detectable sites per protein. Here, we propose a comprehensive quantitative analysis pipeline customized for phosphoproteome data from interventional experiments for identifying key proteins in specific pathways, discovering the protein-protein interactions and inferring the signaling network. We also made an effort to partially compensate for the missing value problem, a chronic issue for proteomics studies. The dataset used for this study was generated using SILAC (Stable Isotope Labeling with Amino acids in Cell culture) technique with interventional experiments (kinase-dead mutations). The major components of the pipeline include phosphopeptide meta-analysis, correlation network analysis and causal relationship discovery. We have successfully applied our pipeline to interventional experiments identifying phosphorylation events underlying the transition to a filamentous growth form in *Saccharomyces cerevisiae*. We identified 5 high-confidence proteins from meta-analysis, and 19 hub proteins from correlation analysis (Pbi2p and Hsp42p were identified by both analyses). All these proteins are involved in stress responses. Nine of them have direct or indirect evidence of involvement in filamentous growth. In addition, we tested four of our predicted proteins, Nth1p, Pbi2p, Pdr12p and Rcn2p, by interventional phenotypic experiments and all of them present differential invasive growth, providing prospective validation of our approach. This comprehensive pipeline presents a systematic way for discovering signaling networks using interventional phosphoproteome data and can suggest candidate proteins for further investigation. We anticipate the methodology to be applicable as well to other interventional studies via different experimental platforms.

## Author Summary

Signal transduction is a ubiquitous and essential mechanism regulating cellular functions, including responses to environmental stress. Dysfunction of signaling pathways results in a variety of diseases, including cancer, diabetes, and cardiovascular disease. Phosphorylation regulates the activity of signaling and target proteins at different cellular locations and controls activation and inactivation of signal pathways. Here, we provide an analysis of phosphoproteome datasets from yeast, utilizing kinase mutants versus wild type strains. In order to provide an objective approach to identify candidate proteins involved in the transition to a filamentous growth form, we proposed and applied a comprehensive pipeline incorporating statistical and mathematical methods to investigate the phosphoproteome data from multiple perspectives. This included phosphorylation variation in response to a single mutant, phosphorylation variation patterns over multiple mutants, and the relationships represented by these patterns. We make an effort to discover the components and targets of the signaling network, infer the network structure, and to find the relationships of changes of protein phosphorylation to cellular functions, specifically in response to stress in the context of filamentous growth.

## Introduction

Cells exchange and receive information from the environment through signaling pathways, which are crucial for cells to maintain normal functions and properly respond to stress and stimuli. Dysregulation of these processes is a major factor in the emergence of many diseases, including cancer, diabetes, and cardiovascular disease. Reversible phosphorylation is one of the major forms of signal transduction and can affect protein function and gene expression [1]–[7]. Investigations into phosphorylation provide insight into signaling pathways by providing the target sites of phosphorylation and the quantitative changes in phosphorylation level in response to genetic or environmental perturbations. Effective, sensitive identification of candidate proteins for further studies remains a challenge in the face of experimental limitations of current technologies which have a high cost component, provide incomplete coverage of the phosphoproteome, and have sampling limitations which affect replicate runs.

Large-scale phosphoproteomics studies on a number of organisms have been carried out using mass spectrometry (MS)-based approaches (reviewed in [8]–[10]). These include two recent global phosphoproteomic studies of the budding yeast (*Saccharomyces cerevisiae*) [5], [6]. In the study carried out by Bodenmiller *at al.* [5], protein kinases and phosphatases were systematically perturbed through gene deletions. The system-wide responses to the perturbations were measured by label-free MS-based quantification, and the results evaluated to determine their contributions to understanding the relationships between these signal transduction proteins and cell pathways. Another global interaction study focused on kinase and phosphatase interactions [6] by capturing protein-protein interactions by affinity capture-immunoblot and identifying the isolated protein complexes by mass spectrometry. These two global studies both adopted label-free, cost-effective quantitative approaches. However, label-free methods typically increase variance relative to isotope enrichment methods [11]. For the purpose of this study, we have used isotope labeled SILAC (Stable Isotope Labeling with Amino acids in Cell culture) method [12], [13] to increase sensitivity to change.

The general scope of this manuscript encompasses a comprehensive pipeline, incorporating statistical and mathematical methods for investigating and evaluating quantitative phosphoproteomic data, the elucidation of candidate proteins, and the identification of processes to be pursued in subsequent molecular biology and genetic studies. The phosphoproteome data utilized in this analysis was obtained from interventional experiments of a subset of yeast kinases involved in filamentous growth. Filamentous growth is a developmental transition observed in *S. cerevisiae* where yeast cells form elongated and connected multicellular filaments; these filaments resemble hyphae but lack the parallel-sided walls and structure of true hyphal tubes. This pseudohyphal growth transition is induced in response to several cell stresses, including nitrogen stress, growth in the presence of short-chain alcohols, and glucose stress [14]–[17]. The filamentous growth form presumably represents a foraging mechanism enabling non-motile yeast to better survive cell stress [14]. During pseudohyphal growth, yeast cells elongate due to a delay in the G2/M transition, exhibit an altered budding pattern, and remain connected after cytokinesis [18], [19]. The resulting pseudohyphal filaments extend superficially from a colony over an agar substrate and invasively downward into the solid substrate below the colony. In liquid culture under inducing conditions, a filamentous strain of yeast exhibits elongated cells and multicellular filaments encompassing typically 3–4 cells. It is important to note that most laboratory strains of *S. cerevisiae* are non-filamentous and that studies of filamentous growth are typically performed in the ∑1278b strain, which we employ here.

**Figure 1:**
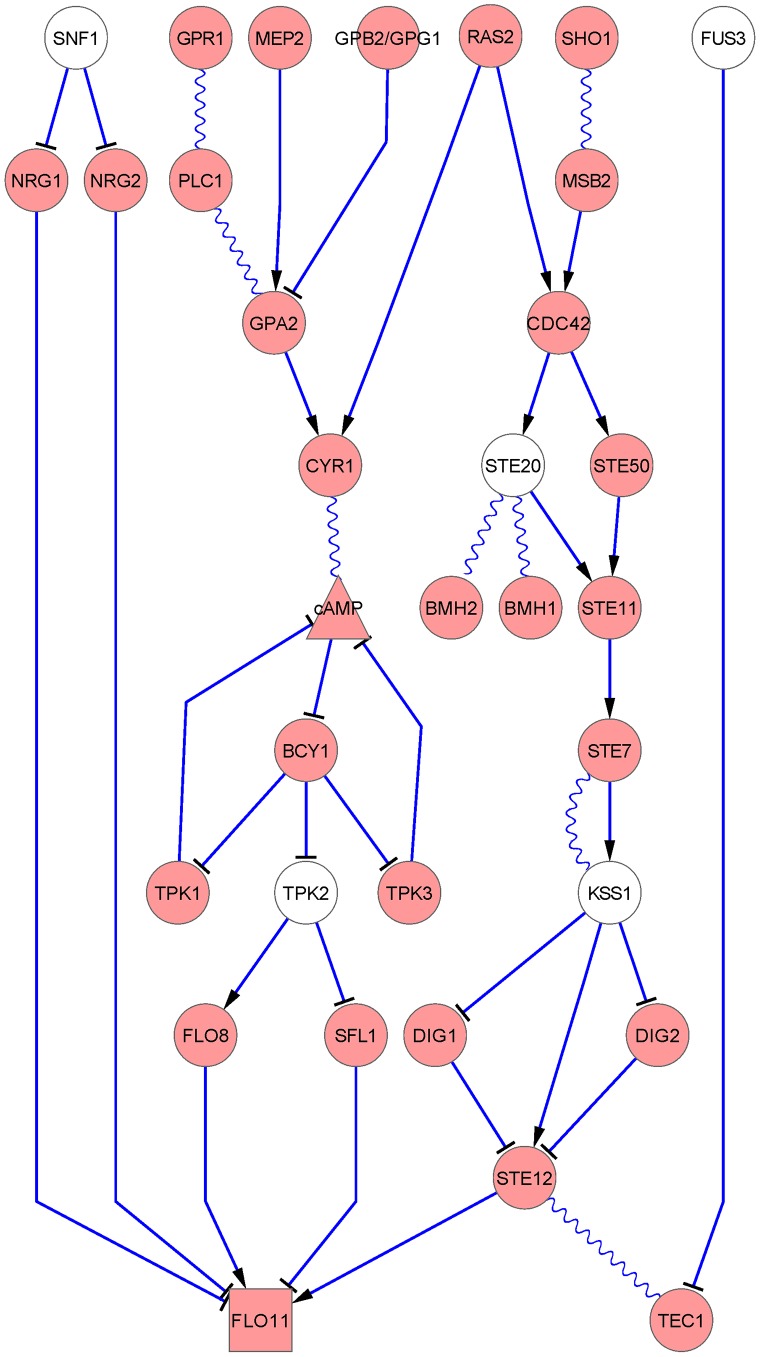
Graphical illustration of the filamentous growth pathway in budding yeast from previous studies. The ellipses are proteins; the rectangles are genes; and the triangles are metabolites. The linkage between shapes: sharp-end arrows indicate stimulation, T-end arrows indicate inhibition, and wavy lines indicate association. The information were extracted from *Science Signaling* Database of Cell Signaling [20] and KEGG database [27]. The white ellipses are five of the eight kinases selected to be mutated in our experiments.

The molecular basis of filamentous growth in *S. cerevisiae* is broad in scope. Classic studies have identified key kinase-based signaling networks that regulate the filamentous growth transition. In particular, yeast filamentous growth is regulated by mitogen-activated protein kinase (MAPK) and protein kinase A (PKA) pathways [15], [20], [21] as well as being impacted by other signaling pathways. MAPK pathways are evolutionarily conserved across phyla and consist of three-kinase cascades serving central roles in signal transduction in eukaryotic cells [22]; the yeast filamentous growth MAPK cascade terminates in the MAPK Kss1p. In *S. cerevisiae*, PKA consists of the regulatory subunit Bcy1p and one of three catalytic subunits Tpk1p, Tpk2p, or Tpk3p; Tpk2p is known to be required for filamentous growth [23]–[25]. It should be noted that the Kss1p MAPK pathway is required for pseudohyphal growth induced by both nitrogen stress and butanol, while the genes *GPR1*, *MEP2*, and *GPA2*, acting upstream of PKA, are not required for butanol-induced filamentous growth [16]. In our experiments, we treated cells with 1% (vol/vol) butanol to induce filamentous growth [26]. A graphical illustration of currently recognized budding yeast filamentous growth pathways, integrating information from authoritative pathway databases and reviews, is shown in Figure 1. While these core signaling units are well defined, the downstream scope of their signaling networks is unclear.

We have generated phosphoproteomic datasets indicating kinase-dependent phosphorylation events underlying the filamentous growth transition. Specifically, we generated kinase-dead mutations (also called kinase-inactivating mutations) for a set of eight kinases that we have identified as components of the yeast filamentous growth response: Ksp1p, Kss1p, Sks1p, Ste20p, Snf1p, Tpk2p, Elm1p and Fus3p [20], [26]–[28]. Each of these kinases exhibits a filamentous growth deletion phenotype, with the deletion of *KSP1*, *KSS1*, *SKS1*, *STE20*, *SNF1*, and *TPK2* yielding a loss of filamentous growth and the deletion of *ELM1* and *FUS3* yielding enhanced filamentation. The kinase-dead alleles of these proteins were constructed by site-directed mutagenesis. The system-wide phosphorylation responses of the mutant strains were determined using SILAC approach, and we used the Mascot search engine [29] followed by MaxQuant software [30] to identify and quantify peptides and proteins. We obtained phosphorylation level changes from the MaxQuant analysis for mutants versus wild type control for the comprehensive quantitative analyses.

The broad focus of the filamentous growth kinase networks in particular has made it difficult to tease out important kinase targets (direct or indirect). Bioinformatics methods provide a promising avenue with which local kinase signaling relationships can be identified. While traditional cluster analyses associated with functional enrichment analysis are useful tools, their performance might be affected by the missing value issue. We need to deal with it in order to obtain reliable clusters and enriched functions. Furthermore, a more integrative and extensive analysis is necessary to find new components of the pathways, uncover relationships between the pathway components, and to elaborate the signaling network structure. Thus we propose this comprehensive quantitative analysis pipeline customized for SILAC data, and partially compensate the missing value issue. The major building blocks include phosphopeptide meta-analysis, correlation network analysis, causal relationship discovery, and validation by literature mining. We have successfully applied the pipeline to analyze our current yeast data. Candidate proteins predicted to contribute to the filamentous growth response were selected by phosphopeptide meta-analysis and correlation network analysis. Causal relationship discovery was performed on candidate proteins identified from our analysis and validated proteins from the literature. The inferred causal relationships, along with the interactions inferred from phosphorylation changes in response to individual mutants, have suggested potential proteins that can be further intervened and studied in the future.

## Results

### Workflow

**Figure 2:**
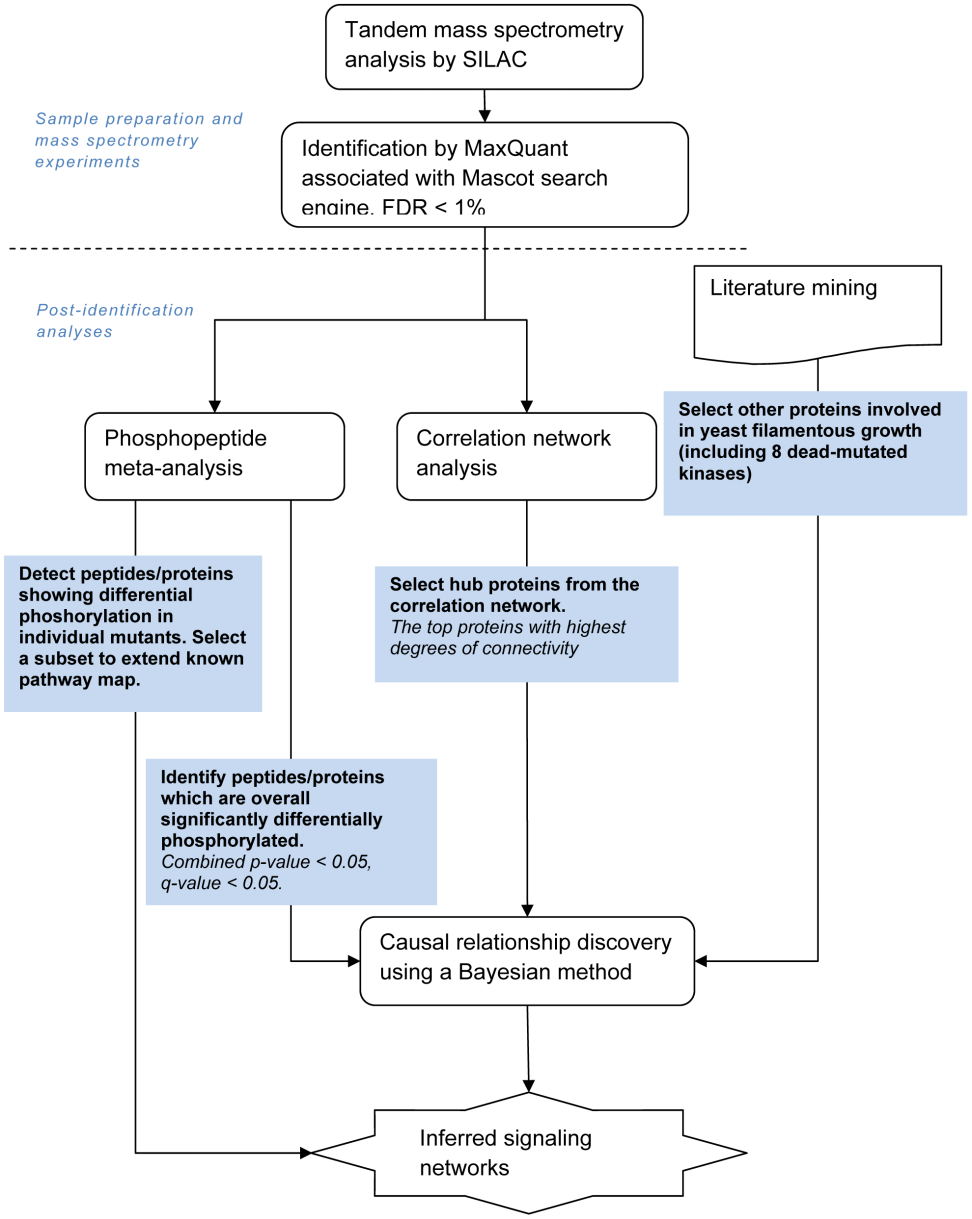
Summary flow chart of the analytical workflow.

An overview of the analytical workflow is shown in Figure 2. Following peptide identification and quantification, the comprehensive post-identification analyses performed consisted of phosphopeptide meta-analysis, correlation network analysis, and literature mining, followed by causal relationship discovery to infer signaling network characteristics. The inferred protein-protein relationships involving hub proteins were backed up by literature, and suggested potential proteins to be intervened in the future studies of yeast filamentous growth pathways. Details of the methodologies are described in **Materials and Methods**. Table 1 lists several important summary numbers of this dataset and subsequent analyses.

**Table 1:** Summary of the dataset and subsequent analyses.

Summary	Number of phosphopeptides	Number of proteins
Identifications in the whole dataset	3,312	1,063
Identifications common among all 8 kinase-dead mutants (KDs)	73	66
Identifications common among 4–8 KDs	882	486
Identifications that are significant in at least 1 KD	863	452
**Globally** significant differential phosphorylation sites	28(5 from complete measurements – high-confidence)	26(5 from complete measurements – high-confidence; 17 have inner connections supported by STRING [53], [54])
High-confidence hub proteins identified from the stringent correlation network	-	19
Proteins known to be involved in filamentous growth from literature mining, and detected in our dataset	-	20(15 of them are significant in at least 1 KD)

### Similar or reciprocal effects induced by kinase-dead mutations

**Figure 3:**
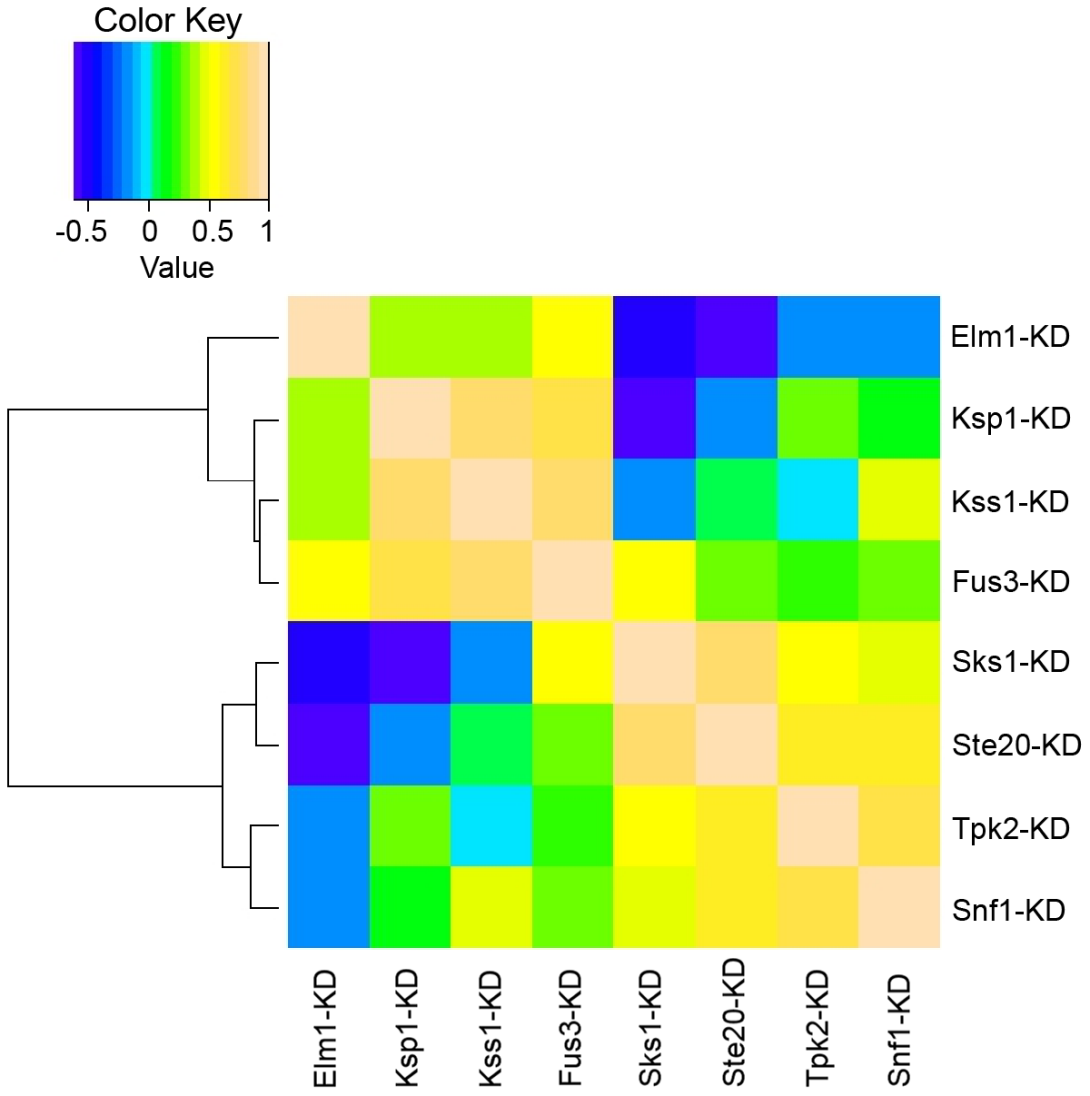
Correlation heat map of the kinase-dead mutants (log2 ratios adopted). The hierarchical clustering tree using Spearman correlation as the similarity metric is drawn along the left side of the heatmap.

The relationships of the eight kinase mutants and their effects on global phosphorylation patterns were subjected to correlation analysis (see ***Overview of the influences inferred from kinase-dead mutations*** in **Materials and Methods**). The results were visualized in a correlation heatmap (Figure 3). The negative correlation between kinase mutants of *SKS1* and *ELM1* are apparent from Figure 3 as are the similarities between some of the mutants (e.g., *SNF1* and *TPK2*). *SKS1* mutants inhibit filamentous growth and *ELM1* promotes it, while *SNF1* and *TPK2* have similar phenotypes. The general correlations between kinases are consistent with their filamentous growth phenotypes and reinforce the identification of core target proteins.

We need to be cautious when interpreting the correlations for partially multiplexed data, such as in triplex SILAC experiments. Because a peptide quantified for one sample is highly likely to be quantifiable for the other two samples in the same triplex, the identification and quantification of phosphopeptides in a triplex experiment tend to be linked. In other words, the overlap within a triplex run should be near 100% but the overlap between different triplex runs will be lower due to instrument sampling limitations. A high number of replicates may help minimize missing data, and compensate for the possible bias introduced by tied identification and quantification; but it is rarely performed due to the high cost of these analyses.

### Phosphopeptide clusters based on phosphorylation changes

A total of 882 phosphopeptides representing 486 proteins were commonly identified in 4–8 kinase-dead (KD) mutants. After the missing values were imputed, the tight clustering method [31] was used to assign those phosphopeptides into groups, and identify the most informative, tight and stable clusters (see ***Clustering phosphopeptides*** in **Materials and Methods**). The results are illustrated in Figure S1 in Text S1. The assignment of proteins and peptides in the top 8 tight clusters is provided in Table 2 and Dataset S1. We also surveyed enriched functions in the tight clusters (Table 2), in terms of functional categories, Gene Ontology, pathways and proteins Domains [27], [32]–[34]. In summary, similar phosphorylation change patterns over multiple mutants (compared to wild type) tends to suggest involvement in similar biological functions. Enriched functional terms include nucleotide phosphate-binding domains, ribosome biogenesis, fructose and mannose metabolism, and glycolysis. Differential carbohydrate metabolism is consistent with the invasive nutrition forage observed under environmental stresses leading to filamentous growth.

**Table 2:** Top 8 tight clusters and functional enrichment.

Cluster	Proteins (traced back from phosphopeptides)	Enriched terms
1	YRO2, BUG1, VPS74, HXK1, PIL1, FBP26, PTK2, NPA3, BIR1, MYO3, UTP14, ARE2, DBP5, RUD3	Nucleotide phosphate-binding region:ATP (P-value = 6.54E-04, Benjamini = 3.4E-2) **
		Nucleotide-binding (P-value = 1.8E-3, Benjamini = 4.2E-2) **
		ATP-binding (P-value = 6.0E-3, Benjamini = 9.3E-2) *
2	VMA2, SEC31, GLY1, PEA2, VTC2, KEM1, UFD1, TIF4631, BCY1, SPA2, MFT1, NEW1, KRE6	-
3	NUP60, SLA1, STU1, YCL020W, VBA4, HOM2, YDR365W-B, VPS74, PSP1, CHD1, NUP145, SPT6, HSE1, ABF1, MEH1, CKI1, YLR413W, SPT5, HRB1, LCB4, CAF20, MRL1	Endosome (P-value = 1.6E-3, Benjamini = 6.6E-2) *
		RNA polymerase II transcription elongation factor activity (P-value = 1.4E-3, Benjamini = 9.6E-2) *
		Transcription elongation regulator activity (P-value = 2.8E-3, Benjamini = 9.9E-2) *
4	FAP7, ITR1, LSB3, LEU1, FLC3, SPT6, YGR125W, CRP1, KEL1, LCB3, YBT1, BDF1, YMR031C, DDR48, YMR295C, GPD2, ZEO1, CAF20, SNF2	-
5	PIN4, CYC8, BUD3, LYS20, CDC34, MAK21, BFR2, SUM1, GLY1, NUP145, PRP43, SPT6, ENP2, YOR1, SSZ1, NUP2, YLR345W, SUB1, ESC1, BDP1, DCP2, RPC31, SLA2, NOP8, ALE1, MSB1, SNU66	Nucleus (P-value = 1.0E-4, Benjamini = 3.4E-3) ***
		Nuclear lumen (P-value = 3.4E-4, Benjamini = 2.7E-2) **
6	SIF2, PPH22, VAC8, HSP12, RTF1, RSC30, TRA1, LCB3, NAP1, SIC1, RPN13, YMR196W, MRE11, MCK1, LEM3, FPK1, LSP1	-
7	IST2, AIM3, RPC53, YDR186C, ECM32, MIG1, HXK2, VHS2, RNR2, UTR1, FBA1, EAP1, YLR257W, PFK2, PFK2, ACC1, YOR052C	Fructose and mannose metabolism (P-value = 3.0E-3, Benjamini = 3.9E-2) **
		Glycolysis (P-value = 1.6E-3, Benjamini = 4.3E-2) **
		Glycolysis/gluconeogenesis (P-value = 9.8E-3, Benjamini = 6.2E-2) *
8	AKL1, IST2, MAK5, FEN1, LHP1, RPC53, SAS10, SHS1, MAK21, DOP1, GCD6, GUK1, CHO1, PDA1, LEU1, NOP7, SPT6, TFG1, HXT1, AIM21, URA2, CDC11, MAK11, VPS13, CBF5, VTA1, CRN1, YMR031C, EFR3, ADE4, NOP12, MAM3, CAF20, PEX25, TIF5	Ribosome biogenesis (P-value = 1.0E-4, Benjamini = 5.0E-3) ***

Functional enrichment P-value and Benjamini-Hochberg corrected p-value (Benjamini) were calculated with DAVID Functional Annotation Tool [33], [34]. They are given in the brackets following corresponding terms.

* Benjamini <0.1,

** Benjamini <0.05,

*** Benjamini <0.01.

All the clusters are highly enriched in the term “phosphoprotein” (not listed above).

We observed examples of multiple phosphorylation domains on the same protein that share similar phosphorylation change patterns and thus end up in the same cluster. For example, “_KGS(ph)FTTELSCR_” (position of the phosphorylated serine: 520) and “_RSS(ph)YISDTLINHQMPDAR_” (position of the phosphorylated serine: 238 or 239) on Psp1p in Cluster 3. It is possible that those phosphorylation sites are co-regulated by the same biological process. They might be closely located in protein tertiary structure or share sequence similarities that allow them to be phosphorylated by the same kinase. Another example where two phosphorylation sites are in the same domain and thus physically close in the protein sequence, “_DQDQSSPKVEVTS(ph)EDEK_” (position of the phosphorylated serine: 495) and “_VEVT(ph)SEDEKELESAAYDHAEPVQPEDAPQDIANDELK_” (position of the phosphorylated threonine: 494) on Leu1p in Cluster 4. Both of these phosphorylation sites were identified in a WT/SNF1/TPK2 experiment, where the serine (S) at position 495 in the former has phosphorylation probability 0.999 (reported by MaxQuant), while the threonine (T) at position 494 in the latter has phosphorylation probability 0.96. These two sites might be alternative phosphorylation sites having similar effects; or the dominancy of either site might be affected by protein cellular localization or kinase activity patterns.

On the other hand, we also found examples of the same protein (e.g., Spt6p) to be clustered in multiple functional groups. Those different sites do not necessarily change phosphorylation in a similar pattern, since they might have different functions and be regulated by different biological processes. All the above observations are worth further investigation.

### Identification of differential phosphorylation in each mutant

**Figure 4:**
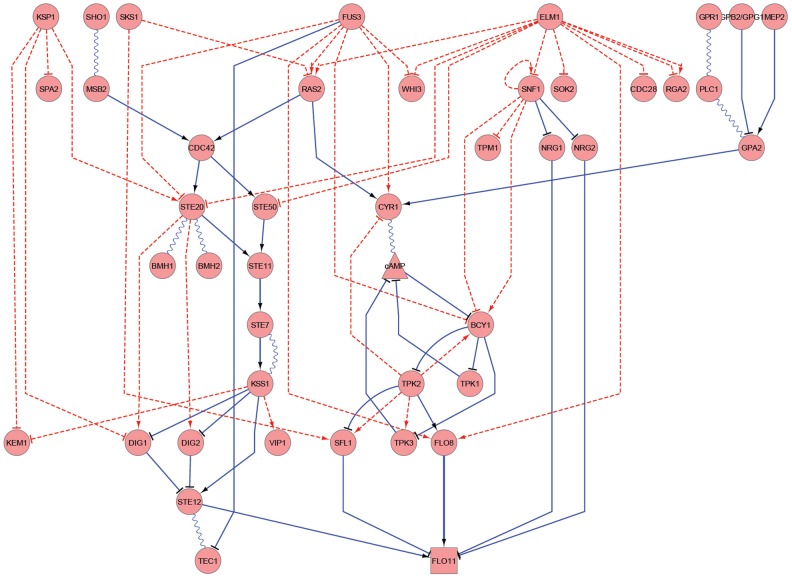
Extended filamentous pathway map. The extended filamentous growth pathway map integrating the known knowledge (Figure 1) and the regulation inferred from significant differential phosphorylation in individual KDs. The inferred regulation might be direct or indirect. The ellipses are proteins; the rectangles are DNAs; and the triangles are metabolites. The linkage between shapes: sharp-end arrows indicate stimulation, T-end arrows indicate inhibition, and wavy lines indicate association. Solid lines indicate physical interactions, while dashed lines indicate changes in phosphorylation.

A total of 863 unique phosphopeptides representing 452 proteins were identified to have significant phosphorylation changes in at least one kinase-dead mutant. We can then infer the downstream proteins regulated by the kinases and the inferred regulation might be direct or indirect. A total of 1,299 significant kinase-phosphopeptide regulation pairs were identified (Dataset S2). We incorporated the corresponding proteins and generated an extended pathway map (Figure 4) based on the known map (Figure 1).

### Phosphopeptides with globally significant phosphorylation changes

A total of 28 phosphopeptides representing 26 proteins from the entire dataset were found to have globally significant phosphorylation changes (Dataset S3). These candidates were picked out without using prior knowledge. The Fisher's probability test [35] was extended to allow missing values (see **Materials and Methods**), and it was used for detecting global significance. Each selected phosphopeptide satisfies the following criteria: the combined p-value < 0.05, q-value < 0.05 for controlling false discovery rate (FDR) [36], and the significance B value < 0.05 in at least 4 out of 8 kinase-dead mutant (KD) versus wild type (WT) conditions. The combined p-value is a measure of global significance, while the significance B value [30] is a measure of significance in an individual experiment. Five of the globally significant phosphopeptides, Nth1p, Hsp42p, Pbi2p, Rcn2p and Pdr12p, were identified with complete measurements (Table 3). We consider them high-confidence candidates. Another adaptively weighted statistic [37] was applied to all complete measurements for validation. Adopting the same selection criterion as above, Nth1p, Pbi2p, Rcn2p and Pdr12 were again identified as globally significant. Both retrospective and prospective validation was performed on selected predictions.

**Table 3:** Globally significant phosphopeptides selected from the complete measurements (high-confidence).

ENSEMBL ID [99]	Standard name	Name description^a^	Modified sequence	Stress response
YDR001C	NTH1	Neutral trehalase;Alpha,alpha-trehalase;Alpha,alpha-trehalose glucohydrolase	_RGS(ph)EDDTYSSSQGNR_	Nth1p is a multiple stress responsive protein [38], [57].
YNL015W	PBI2	Protease B inhibitors 2 and 1;Proteinase inhibitor I(B)2	_HNDVIENVEEDKEVHT(ph)N_	Pbi2 gene deletion leads to decreased resistance to hyperosmotic stress [58].
YOR220W	RCN2	Regulator of calcineurin 2;Weak suppressor of PAT1 ts protein 1	_NKPLLSINT(ph)DPGVTGVDSSSLNK_	Rcn2p is Induced in response to DNA-damaging agent methyl methanesulphonate [59].
YPL058C	PDR12	ATP-dependent permease PDR12	_HLSNILS(ph)NEEGIER_	Pdr12 is strongly induced by weak acid stress [60] and is a target of the transcription factor War1p [61] which elicits weak organic acid stress adaptation through active efflux [62], [63].
YDR171W	HSP42	Heat shock protein 42	_KS(ph)S(ph)SFAHLQAPSPIPDPLQVSKPETR_	Protein expression is induced by stresses such as heat shock, salt shock and starvation [52].

a Annotated with MaxQuant.

Nth1p is a key enzyme in the trehalose pathway which plays a crucial role in glucose homeostasis and stress responses [38], [39] and is a substrate phosphorylated for both Tpk1p and Tpk2p [40]. The *NTH1* gene also has been reported to have genetic interactions with the *TPK1* and *TPK2* genes [41]. It has been reported to physically interact with the kinase Sks1p [1] and with Bmh1p [42]. The above direct interactors of Nth1p, *i.e.*, Tpk1p, Tpk2p, Sks1p and Bmh1p, are all known to play roles in filamentous growth [26], [43]–[47]. The Rcn2p protein was also reported to physically interact with Bmh1p [42], which associates with the Ste20p protein involved in filamentous growth [47], [48]. Bmh1p may also interact with Tpk1p [49]–[51]. Thus, Nth1p and Rcn2p have been closely associated with a number of proteins known to be involved in filamentous growth. Hsp42p has a physical association with Fus3p [42], and its expression is induced under starvation [52]. The remaining two proteins in Table 3 have not yet been closely linked to filamentous growth but play roles in other stress responses and represent new leads.

We also searched the STRING database [53], [54] to investigate the inner connections between the 26 globally significant proteins (shown in Figure S2 in Text S1). STRING assigns the confidence of protein-protein interactions integrating high-throughput experiments, genetic context, co-expression and other previous knowledge. In Figure S2 in Text S1, 17 proteins, including Nth1p, Hsp42p, Rcn2p, Pbi2p, Hsp26p, Bfr1p, YGR250C protein, Leu1p, Lys20p, Cdc19p, Fol2p, Pil1p, Abp1p, Cdc11p, Shs1p, YLR413W protein and Pxr1p, have direct or indirect connections. It presents a closely inter-connected sub-network embodying Nth1p, Pbi2p, Rcn2p, Hsp42, YGR250C protein and Hsp26p.

### Correlation network

**Figure 5:**
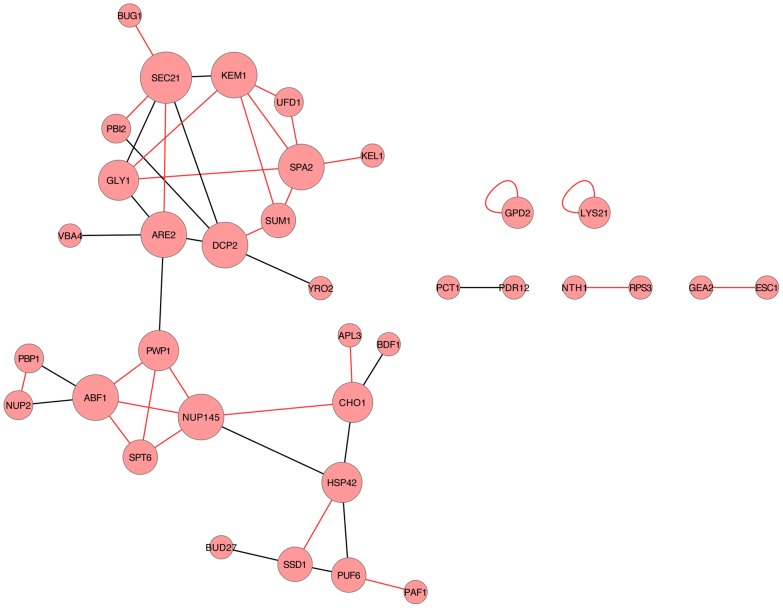
Stringent correlation network of phosphoprotein pairs. Red lines indicate positive correlations, while black lines indicate negative correlations. The larger the node size, the greater the degree of connectivity.

All possible pairs among the 73 common phosphopeptides with complete measurement were tested using the Pearson correlation. A total of 45 strongly correlated phosphopeptide pairs were identified, each satisfying the following criteria: the correlation test p-value < 0.05, and the stringent requirement of |Pearson correlation coefficient| ≥ 0.9. Detailed information on the 45 pairs of phosphopeptides is provided in Dataset S4. Twenty-seven of the pairs have positive correlations, while 18 pairs have negative correlations. A stringent protein correlation network containing 35 proteins (Figure 5) was generated by connecting the strongly correlated peptide pairs and then tracing the peptides back to their parent proteins.

### Identifying core-components in the correlation network

In the protein correlation network, proteins with the highest degrees of connectivity are considered core components in the network. The 19 proteins having degrees greater than 1 (protein self-connection ignored) in the stringent protein correlation network were predicted to be core components of the network. Detailed descriptions and evidence of the proteins are summarized in Table S2 in Text S1. Kem1p, Spa2p and Spt6p have been reported to be directly involved in filamentous growth in previous literature. Six other proteins, Are2p, Dcp2p, Hsp42p, Ssd1p, Sum1p, and Ufd1p, have reported evidence in terms of genetic and/or physical interactions with known components of filamentous growth. The remaining proteins have been implicated in various stress responses, including the unfolded protein response (e.g., sensitivity to tunicamycin), osmotic shock, and thermal shock, but not previously linked to filamentous growth. Pbi2p has not been reported previously as being involved in filamentous growth; however, our experimental results indicate that a haploid strain of *S. cerevisiae* deleted for *PBI2* exhibits decreased invasive growth relative to wild type (see ***Experimental validation*** in **Results**).

Gpd2p and Lys21p are two self-connected proteins. The self-connection was built up by two distinct phosphorylation sites on the protein. Gpd2p has not been related to filamentous growth in *Saccharomyces cerevisiae*. Its homolog Gpd2p in *Candida albicans*, is involved in core stress responses, and *GPD2* is induced upon pseudohyphal growth *in S. cerevisiae* [42]–[49].

### Literature mining

In addition to the candidate proteins predicted from our dataset, we retrieved from the literature and authoritative databases [20], [26]–[28], [55], [56] a list of proteins involved in filamentous growth. A total of 69 unique proteins, not all being phosphoproteins, were extracted (Table S1 in Text S1), and 20 of them have been detected in our phosphoproteome dataset. Among those, 15 proteins, including Bcy1p, Cdc28p, Cyr1p, Dig1p, Dig2p, Flo8p, Kem1p, Ras2p, Sfl1p, Snf1p, Spa2p, Ste20p, Ste50p, Tpk3 and Tpm1p, showed significant phosphorylation changes in at least one kinase-dead mutant, and are displayed in our extended pathway map (Figure 4).

### Causal Bayesian network

The interactions retrieved from the differentially phosphorylated proteins in individual kinase-dead mutants (the dashed edges in Figure 4) did not make use of phosphorylation change pattern over different kinase-dead mutants, and the protein pairs must contain a mutated kinase. In contrast, the correlation network is a network of the common peptides, taking into account the protein responses in all the kinase-dead mutants, and the correlated protein pairs do not necessarily contain the mutated kinases. Note that this network is not directed and more information may be gleaned from a causal analysis. We implemented causal relationship discovery to detect the direction of influences between proteins with the understanding that the relationships may be direct or indirect. A total of 46 unique proteins, including the kinases we mutated, the predicted high-confidence globally significant proteins and hub proteins, and other literature reported proteins, were selected to construct the network. All are listed in Table 4.

**Table 4:** Focus proteins used for causal relationship discovery.

Mutated kinases	Globally significant (high-confidence)	Hub proteins (high-confidence)	From literature mining and detected in our dataset
	(also see Table 3)	(also see Table S2 in Text S1)	(also see Table S1 in Text S1)
KSP1	NTH1	SEC21	BCY1
KSS1	PBI2	ABF1	BMH1
SKS1	RCN2	ARE2	BUD2
STE20	PDR12	DCP2	CDC28
SNF1	HSP42	KEM1	CYR1
TPK2		NUP145	DIG1
ELM1		SPA2	DIG2
FUS3		CHO1	FLO8
		GLY1	GPR1
		HSP42	KEM1
		PWP1	NRG1
		PUF6	PEA2
		SPT6	RAS2
		SSD1	SFL1
		SUM1	SNF1
		NUP2	SPA2
		PBI2	STE20
		PBP1	STE50
		UFD1	TPK3
			TPM1

**Figure 6:**
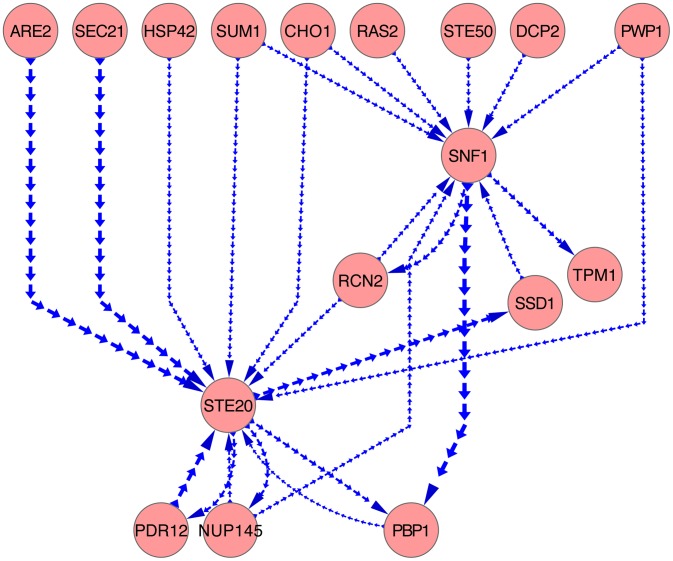
Causal Bayesian network. Each edge indicates a potential causal influence between proteins, which might be a direct or indirect influence. It does not distinguish activation and inhibition. The thicker the edge, the higher the posterior probability.

Bayesian network modeling identified causal influences for 22 protein pairs (44 phosphopeptide pairs) (Table S3 in Text S1), satisfying the posterior probability of the relationship greater than 0.5. The network comprising all the causal relationships is presented in Figure 6. Among those, only 6 protein pairs have the posterior probability higher than 0.7. The other protein pairs do not have high probability since the samples available for training the model is limited due to the missing data issue caused by instrument limitation. The arrows in Figure 6 only indicate the existence of causal influence, but do not specify whether the influence is activation or inhibition. The causal relationship discovered might be between proteins that are not immediately adjacent in pathways so the relationship could be quite indirect. For example, the causal relationship between Rcn2p and Ste20p might be indirect: Rcn2p and Bmh1p have physical interaction captured by affinity capture-MS [42], while Bmh1p associates with Ste20p to influence filamentous growth [47].

Through another inspection of the phosphorylation change patterns of the peptide pairs detected with relatively strong causal influences (posterior probability higher than 0.7), we observed that: Ste20p has opposing phosphorylation changes compared to Are2p, Pdr12p and Sec21p; two phosphopeptides (the same amino acid sequence but different phosphorylation sites) on Hsp42p present opposing phosphorylation changes compared to Ste20p; and Pbp1p presents consistent phosphorylation change compared to Ste20p. With caution we predict that the opposing pattern implicates an inhibitive influence of Are2p, Pdr12p and Sec21p to Ste20p; and similarly, inhibition of Hsp42p to Ste20p; while Pbp1p shed activating influence to Ste20p. Again, we emphasize that the influence might be quite indirect and even be influenced by multiple pathways.

### Experimental validation

**Figure 7:**
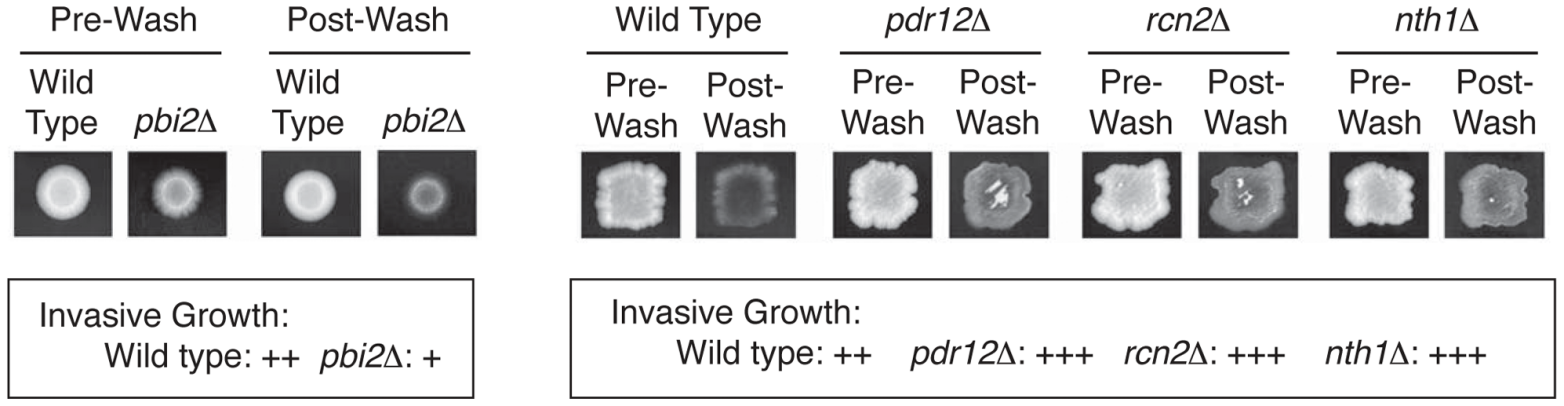
Phenotypic analysis of genes predicted to contribute to the yeast filamentous response. The genes *PBI2*, *PDR12*, *RCN2*, and *NTH1* were deleted in a haploid strain of the filamentous ∑1278b genetic background by targeted replacement with the G418-encoding *kanMX6* cassette. The resulting strains were grown 3–4 days under normal growth conditions, and invasive growth was assayed in these strains and in a wild-type strain according to standard protocols using a plate-washing assay. Deletion of *PBI2* resulted in decreased invasive growth upon plate washing, and strains deleted for *PDR12*, *RCN2*, and *NTH1* yielded hyperactive invasive growth. The increased invasive growth in these strains was most clearly evident in patched cultures as shown.

Our computational analyses highlight the proteins Nth1p, Pbi2p, Pdr12p, and Rcn2p as undergoing globally significant phosphorylation changes. To determine if these proteins do in fact impact filamentous growth in *S. cerevisiae*, we constructed haploid strains singly deleted for each gene and assayed for filamentation phenotypes. Precise gene deletions were carried out using a standard PCR-based strategy, and resulting haploid strains were assayed for invasive growth by standard plate-washing assays under normal growth conditions [17]. Invasive growth, characterized by filament penetration into the agar substrate, was decreased upon deletion of *PBI2* relative to wild-type. In addition, the deletion of *NTH1*, *PDR12*, and *RCN2* yielded exaggerated invasive growth relative to an otherwise isogenic wild-type strain. Results are shown in Figure 7. All four proteins have been previously implicated in various yeast stress responses, but not specifically with respect to filamentous growth [38], [52], [57]–[63]. Nth1p, *i.e.* neutral trehalase, is involved in the trehalose pathway, which is a glucose storage pathway [64]. Pbi2p is a cytosolic inhibitor of vacuolar proteinase B, and is involved in the regulation of proteolysis [65]–[69]. Rcn2p, a regulator of calcineurin [70], is induced in response to DNA-damaging agents [59]. Pdr12p is a multidrug transporter inducible by weak acid, and is required for weak organic acid resistance [71], [72]. These four proteins are not reported to be signaling molecules themselves, but we demonstrate that they appear to play roles in filamentous growth and are likely downstream targets of the filamentous growth pathways.

In summary, all four deletions result in differential invasive growth compared to the wild type control, providing prospective validation for our approach to identification of candidate proteins in this biological system from phosphoproteomics data alone.

## Discussion

In this study, we demonstrate that interventional phosphoproteome studies can provide new insight into signaling pathways involved in biological processes such as yeast filamentous growth. In order to increase sensitivity to smaller changes in phosphorylation relative to previous yeast global phosphoproteome studies [5], [6], we used SILAC, an isotope labeling approach. Isotope labeling approaches are generally more precise relative to label-free approaches [11], but require greater resources to implement, resulting in trade-offs between precision and missing data due to sampling limitations inherent to current instruments. We proposed and developed a comprehensive computational and statistical analysis pipeline for the post-identification studies of phosphoproteome data. The analyses are aimed at discovering candidate components of significant pathways involved in filamentous growth as well as the potential targets of the pathways, and to provide more information on the signaling network structure by monitoring changes in phosphorylation in response to mutational interventions. We applied the pipeline to analyze our interim high mass accuracy yeast phosphoproteome datasets and a total of 882 unique phosphopeptides representing 486 proteins were identified as significantly influenced by at least one out of 8 kinase-dead mutants. Twenty-eight unique phosphopeptides having globally significant phosphorylation were identified from the whole dataset among which 5 peptides representing 5 proteins, Nth1p, Pbi2p, Rcn2p, Pdr12p and Hsp42p, were identified as high-confidence candidates. Nineteen candidate proteins with relatively high degrees of connectivity were selected as hub proteins in the stringent correlation network (Pbi2p and Hsp42p were identified as hub proteins too). Among the high-confidence candidate proteins, 3 proteins have been previously reported to be directly involved in filamentous growth and another 6 proteins were also supported, in terms of genetic and physical interactions with known components involved in filamentous growth. The remaining proteins have been implicated in various other stress responses and may play roles in filamentous growth or may be secondary stress responders. In particular, we validated four candidate proteins, Nth1p, Pbi2p, Pdr12p and Rcn2p as impacting invasive growth. Causal relationship discovery was further performed on the candidates and validated proteins. The inferred causal relationships, along with the interactions inferred from phosphorylation changes in response to individual mutants, form phosphoprotein interaction networks, which suggested potential proteins to be intervened in future studies.

Each of the kinases mutated in this study had previously been implicated in filamentous growth. Many of these kinases are known to also affect pathways that are not involved directly in filamentous growth. However, the proteins which change phosphorylation level in response to multiple mutants are reasonable candidates involved in filamentous growth. The sensitivity of such detection is constrained by the degree of overlap between pathways, the coverage of pathways by the mutants, and the extent of missing data. Upstream components of isolated pathways may be missed, while downstream core components are more likely to be identified.

A remaining challenge for quantitative phosphoproteome analysis arises from the sampling limitations and resolution of current mass spectrometers [11]. This feature of tandem mass spectra of complex mixtures results in poor overlap of peptides identified across samples unless a relatively large number of replicate experiments are carried out (which is time consuming and often economically impractical for large-scale projects). For this reason, a significant number of missing values exist in these datasets which can obscure potential candidates for further validation studies. This is likely to be alleviated to some extent in the future as mass spectrometry technologies continue to improve, but we have developed methods to partially compensate for the missing data issue. In the phosphopeptide meta-analysis, an extension of Fisher's combined probability test was made to relax the restrictions of complete measurements. The causal network modeling component was also developed to allow missing values without excluding the incomplete measurements. We also performed cluster analysis of phosphopeptides. Instead of adopting traditional clustering methods, we directly identified the most stable clusters using missing value-imputed data. Our approach was able to pick out significantly enriched functions, and identify a number of reliable candidate proteins for further validation of which four were validated.

This analysis pipeline has been developed to study yeast filamentous growth pathways; however, the methodology is not limited to yeast or this biological process. It can be applied to other complex organisms to facilitate investigation into various biological processes. We anticipate the methodology to be applicable as well to other interventional studies via different experiment platforms.

## Materials and Methods

### Mass spectrometry data

Tandem mass spectrometry data were generated from a series of triplex SILAC [12], [73], [74] experiments of kinase-dead mutant (KD) strains versus the wild type (WT) haploid filamentous yeast ∑1278b strain. Eight yeast kinases, KSP1p, KSS1p, SKS1p, STE20p, SNF1p, TPK2p, ELM1p and FUS3p, all known to be involved in filamentous growth [20], [26], [27], were chosen to generate kinase-dead mutations (inactivated alleles) individually. We investigated the yeast phosphoproteome from the eight mutants vs. wild type. We have obtained 2–3 replicates for 7 (out of 8) kinase-dead mutants. The dataset constitution is listed in Table S4 in Text S1. Because mass spectrometry experiments are time-consuming and costly, most recent studies in proteome research perform two [75], [76] or three replicates [77], [78] which contributes to the missing data problem in proteomics.

All strains were auxotrophic for Lys and Arg, and were grown on defined medium supplemented with the appropriate isotopic forms of Lys and Arg. The cultures were grown to log phase, and treated with 1% (vol/vol) butanol to induce filamentous growth [26]. The treated samples were incubated for another 16 hours to obtain enough proteins for mass spectrometry analysis. The final O.D. at 600 nm reached a high value usually between 1.0 and 1.5. Different Lys and Arg isotope forms were used to label the three samples in a triplex SILAC experiment: light (Lys0/Arg0) for WT control sample, medium (Lys4/Arg6) and heavy (Lys8/Arg10) for two different mutant samples. Cells were harvested by centrifugation and lysed in the presence of protease and phosphatase inhibitors. In SILAC experiments, samples were pooled at the harvest stage before protein extraction. Samples pooled at this early stage can reduce both systematic and random errors that may occur in later sample preparation [79], [80], thus the results have smaller variance compared to unpooled samples. Small sample sizes (two or three replicates) is acceptable for the low-variance SILAC experimental design. We observed in our SILAC experiments that the majority of the ratio “variability” in the data was less than 20. (The “variability” is reported by MaxQuant and is defined as the standard deviation of all log ratios used for obtaining the reported ratio value multiplied by 100 [30], [81].)

Protein levels were determined by the Bradford protein assay and the proteins from the triplex labeling were then pooled, and were digested by trypsin. The digest was separated into fractions using strong cation-exchange (SCX) fractionation, followed by selective enrichment of phosphorylated peptides using titanium dioxide [82], [83] and then analyzed by LC-MS/MS using a Thermo Fisher Orbitrap XL mass spectrometer. Peptides were identified using MaxQuant software [30] following the Mascot search engine [29], and filtered requiring peptide identification FDR<1%. For Mascot searches, enzyme specificity was set to trypsin. Carbamidomethyl cysteine was set as a fixed modification. N-terminal carbamyl, oxidized methionine, as well as phosphorylation of serine, threonine, and tyrosine were set as variable modifications. Some missed cleavage was observed and could potentially contribute to the variance. The method for calculating peptide identification FDR based on concatenated databases was described by Cox J and Mann M [30]. A total of 3,312 phosphopeptides representing 1,063 proteins were identified. Among those, 73 unique phosphopeptides representing 66 common proteins were commonly identified in all the 8 kinase-dead mutants; while, 882 phosphopeptides representing 486 proteins were common to at least half of the kinase-dead mutants.

### Post-identification analyses

#### Post-identification analyses

In the meta-analysis, we contrast and combine the results from different KD-versus-WT experiments, so that to find the correlations between kinase-dead mutants, categorize peptide phosphorylation patterns over experiments, and identify differentially phosphorylated peptides.

##### Overview of the influences inferred from kinase-dead mutations

The relative phosphorylation level obtained for each phosphopeptide is represented as a ratio for each of the 8 kinase-dead mutants (KD) versus wild type (WT) under filamentous growth conditions. Two examples of phosphopeptides identified in all 8 kinase-dead mutants are shown in Table 5. The ratio lists of all the identified phosphopeptides are aligned to constitute a ratio matrix. The quantity measuring statistical significance of each ratio, *i.e.*, the significance B value, was calculated with MaxQuant [30]. The ratios shown in Table 5 were extracted before filtering by statistical significance.

**Table 5:** Ratio lists for two representative phosphopeptides from the ratio matrix.

Phosphopeptide	Phosphorylation fold-changes in following KD-vs-WT conditions							
	Sks1-KD vs. WT	Ste20-KD vs. WT	Snf1-KD vs. WT	Tpk2-KD vs. WT	Elm1-KD vs. WT	Fus3-KD vs. WT	Kss1-KD vs. WT	Ksp1-KD vs. WT
ADDEEDLS(ph)DENIQPELR	0.72	0.71	0.70	0.52	1.0	0.88	0.83	0.86
ADGTGEAQVDNS(ph)PTTESNSR	2.3	3.7	2.1	2.2	0.33	0.58	0.75	0.69

Phosphorylation level of each phosphopeptide is represented in a list of ratios. We used the peptide ratios provided by the MaxQuant output, which have been normalized for each LC-MS/MS run [30]. The significance B values provided by MaxQuant are not shown here. For the cluster analysis, if a phosphopeptide is detected multiple times under the same KD-versus-WT condition, the median of all its ratios are taken. S(ph) or T(ph) indicates that the specific amino acid, serine or threonine, is phosphorylated, respectively.

For the purpose of evaluating similar or reciprocal effects on phosphorylation changes in response to different kinase mutations, we generated a correlation heatmap of the kinase-dead mutants (see Figure 3), which is presented as Spearman correlations between pairs of mutants. In order to avoid the strong correlation dominated by the majority of peptides whose phosphorylation do not change significantly, only the peptides having at least 2-fold changes in both mutants were used for calculation. Positive or negative correlations can be interpreted as similar or reciprocal effects on phosphorylation induced by different kinase mutations.

##### *Clustering phosphopeptides*

Our goal of this cluster analysis is to find the groups of phosphopeptides sharing similar phosphorylation change patterns, which are likely to be involved in similar functional pathways. The phosphopeptides commonly identified in 4–8 KD-versus-WT conditions were selected, and the missing values were imputed (on log2 scale) using 5-nearest neighbor averaging [84], [85]. The imputed dataset was analyzed using the tight clustering method [31], which sequentially identified the most informative, tight and stable clusters from the data, without enforcing all peptides to be clustered.

We also attempted several traditional clustering methods, including hierarchical clustering methods [86] and PAM (Partitioning Around Medoids) [87]. These methods enforce clustering for all peptides. However, due to the presence of large numbers of scattered peptides that do not tightly match to any pattern, it is difficult to determine the number of clusters and discover stable functional clusters. In contrast, the tight clustering method is more suitable for data with scattered peptides. It identifies the tight clusters in decreasing order of stability, and the number of clusters is less crucial [31].

Note that the cluster analysis was performed at the peptide level rather than the protein level, because many proteins contain multiple phosphorylation domains whose responses may correlate or not, depending on the function of phosphorylation at those sites and the physiological conditions examined. Protein identities were traced back from the peptide identifications while accounting for protein isoforms.

##### Functional annotation within each tight cluster

The functional terms were annotated for the proteins in top tight clusters to survey functional enrichment. The Functional Annotation Tool on DAVID v6.7 [33], [34] was used to facilitate annotation.

##### Identification of differential phosphorylation in each mutant

The phosphopeptides that change phosphorylation level significantly in each individual KD-versus-WT experiment were selected by the significance B value < 0.05.

##### Identification of globally significant differential phosphorylation

The kinases selected to be dead mutated are all known to be involved in filamentous growth. The proteins which have globally significant responses in the mutants versus WT controls are potential components involved in filamentous growth or expression products of the gene targets. Detecting globally differentially phosphorylated peptides combining the results from all the KD-versus-WT experiments is a multiple testing problem [88]. Due to the missing data issue common in proteome data, it is too stringent and impractical to require a candidate to be completely significant in all the experiments. Thus, we relax the requirement, and use less stringent methods which can still identify the candidates having global significance. We extended the Fisher's combined probability test [35] to allow missing values, and it was applied to solve the multiple testing problem.

In the framework of Fisher's method, the two-tailed p-value 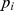 for an individual significance test in a KD-versus-WT experiment is calculated as twice the significance B value. In our dataset, 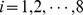, corresponding to the 8 KD-versus-WT conditions, and the total number of individual tests, 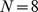. The test statistic 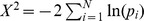 follows a chi-square distribution with 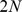 degrees of freedom. Thus, the p-value for the test statistic 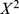 can be determined, which is the combined p-value for all 8 individual tests. Each identified phosphopeptide has a combined p-value as a measure of global significance. The extension of Fisher's method: for each phosphopeptide, its combined p-value was calculated from all of its available significance B values. All the non-missing values were retained for calculating the combined p-value, rather than excluding the incomplete data from the dataset. The FDR of multiple testing is controlled using the *Benjamini-Hochberg* procedure [36].

We also adapted an adaptively weighted statistic-based method (missing values not allowed) [37], which was initially developed for detecting differential gene expression, for detecting differential phosphorylation from our common peptides appearing in all KDs. The globally significant phosphorylation sites detected by these two methods were generally consistent.

##### *Correlation network analysis*

A correlation network of all the 73 common phosphopeptides with complete measurements was generated based on their phosphorylation changes under all 8 KD-versus-WT conditions. The Pearson correlation coefficient between each pair of distinct phosphopeptides was calculated. Strong correlations meet the following criterion: p-value of the Pearson's correlation test < 0.05, and a stringent requirement of |Pearson correlation coefficient| ≥ 0.9. The protein identifications can be traced back from the phosphopeptides.

The correlation network among proteins is an undirected network. Degrees of connectivity for each protein in the network can provide an assessment of importance of the protein. The higher the degree, the more frequently the protein is involved in interactivities with other proteins in the network. From this measurement, we predict core-components in the correlation network.

##### *Literature mining*

In addition to the candidate proteins predicted by global differential phosphorylation and the core-components identified from the correlation network, we also retrieved a list of proteins reported as known or potential components involved in filamentous growth from literature as well as authoritative databases, such as SGD [55], [56], BIOGRID [89] and *Science Signaling* Database of Cell Signaling [20]. Note that people have usually used different terms to refer to filamentous growth in haploid cells; “filamentous growth,” “filamentation” might all refer to the same biological process. In SGD database, we search both key words for Descriptions and GO Biological Process terms associated with the proteins.

##### *Causal Bayesian network modeling*

The correlation network is intuitive; however, it is not directed, and direction information for networks is quite useful for interpretation. For this reason we went beyond correlation analysis to causal Bayesian network modeling. Because different phosphopeptides from the same protein do not definitely change phosphorylation level in the same direction, the network modeling must be performed on peptide level, and then traced back to their parent proteins.

##### Data preprocessing

If a phosphopeptide was detected more than once in a specific mutant, the median of the fold-changes was taken as a representative of the response in this mutant. The phosphorylation fold-changes of peptides were discretized into three states based on the 2-fold change criterion [90]: if the ratio is smaller than 0.5, the state is categorized into *under-phosphorylation*; if the ratio is greater than 2, the state is categorized into *over-phosphorylation*; otherwise, the state is categorized into *baseline*.

##### Causal relationship discovery

A causal Bayesian network is a Bayesian network in which a directed edge is interpreted as a causal influence from the parent node to the child node [91], [92]. In our study, each protein (represented by unique phosphopeptides) is considered as one node in the network, and a directed edge starting from the node of protein X pointing to the node of protein Y represents a causal influence of protein X on Y. Disregarding confounding influences, there are three simple model structures between two proteins X and Y: (1) X has causal influence on Y; (2) the opposite; (3) no causal relationship between X and Y. Note that the directed edge only indicates the direction of causal influence, but do not tell whether the influence is activation or inhibition.

Non-informative prior distribution of the model structures is used. For given data, 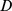, and prior knowledge, 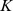, we want to find the model structure, 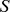, that has the highest posterior probability, 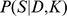. According to Bayes' theorem, 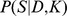 ∝ 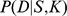. While all the nodes have been discretized in ***Data preprocessing***, assuming the causal mechanisms are local and independent, and the prior distribution of the parameters associated with each node is Dirichlet, the marginal likelihood 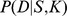 can be obtained by the Bayesian Dirichlet equivalent (BDe) metric [91], [93]–[95]. For the mixture of observational and interventional data, only the passively observed cases are counted in the BDe metric calculation [91], [95]. The model structure with the highest posterior probability is assigned to the corresponding pair of proteins.

The analyses were implemented in R v2.15.1 and MATLAB R2012a. The causal Bayesian network structure learning was performed in MATLAB using BNT (Bayes Net Toolbox for MATLAB) v1.0.7 [96]. Cytoscape v2.8.3 [97], [98] was used for network visualization.

## Supporting Information

Dataset S1 **The top 8 tight clusters.**
(XLSX)

Dataset S2 **Significantly differentially phosphorylated peptides in individual kinase-dead mutants.** This is a non-redundant list. Based on our observation, the kinase-phosphopeptide pairs are at least as significant as the significance B value presented in the table.
(XLSX)

Dataset S3 **Detailed descriptions of 28 globally significant phosphopeptides selected by using an extended Fisher's strategy on all identified phosphopeptides.**
(XLSX)

Dataset S4 **Strongly correlated phosphopeptide pairs (from complete measurements, p<0.05 and |correlation| > = 0.9).**
(XLSX)

Text S1 **Supplementary file.** This pdf file contains the supplementary Tables S1, S2, S3, S4 and Figures S1, S2. The supplementary Datasets S1, S2, S3, S4 are provided in separate excel files.
(PDF)
