# Supplementary material for: Towards Systematic Discovery of Signaling Networks in Budding Yeast Filamentous Growth Stress Response Using Interventional Phosphorylation Data
Source: PLoS Comput Biol. 2013 Jun 27;9(6):e1003077. doi: 10.1371/journal.pcbi.1003077 (PMC3694812; doi:10.1371/journal.pcbi.1003077)
Supplement: Text S1 — Supplementary file. This pdf file contains the supplementary Tables S1, S2, S3, S4 and Figures S1, S2. The supplementary Datasets S1, S2, S3, S4 are provided in separate excel files. (PDF) [file pcbi.1003077.s005.pdf]

Towards Systematic Discovery of Signaling Networks in Budding Yeast Filamentous Growth Stress Response Using Interventional Phosphorylation Data

Supplementary file

This pdf file contains the supplementary Tables S1-S4 and Figures S1-S2.

The supplementary Datasets S1-S4 are provided in separate excel files.

Supplementary Tables

Table S1. Proteins involved in filamentous growth obtained from literature and databases.

	Standard.name	ENSEMBL.ID	Detected in our dataset?
1	BCY1	YIL033C	YES
2	BMH1	YER177W	YES
3	BMH2	YDR099W	
4	BUD2	YKL092C	YES
5	CAP2	YIL034C	
6	CDC28	YBR160W	YES
7	CDC42	YLR229C	
8	CYR1	YJL005W	YES
9	DFG10	YIL049W	
10	DFG5	YMR238W	
11	DIG1	YPL049C	YES
12	DIG2	YDR480W	YES
13	DLD3	YEL071W	
14	DOT6	YER088C	
15	DSE1	YER124C	
16	ECM25	YJL201W	
17	ELM1	YKL048C	
18	EMP70	YLR083C	
19	FIG2	YCR089W	
20	FLO10	YKR102W	
21	FLO8	YER109C	YES
22	FUS3	YBL016W	
23	GAS1	YMR307W	
24	GPA2	YER020W	
25	GPR1	YDL035C	YES
26	GRE2	YOL151W	
27	HKR1	YDR420W	
28	HMS1	YOR032C	
29	HMS2	YJR147W	
30	HSL1	YKL101W	
31	ILM1	YJR118C	
32	KEM1	YGL173C	YES
33	KSP1	YHR082C	
34	KSS1	YGR040W	
35	MEP2	YNL142W	
36	MGA1	YGR249W	
37	MSB2	YGR014W	

38	MUC1	YIR019C	
39	NRG1	YDR043C	YES
40	NRG2	YBR066C	
41	PEA2	YER149C	YES
42	PLC1	YPL268W	
43	PTP1	YDL230W	
44	RAS2	YNL098C	YES
45	SEC66	YBR171W	
46	SEM1	YDR363W-A	
47	SFL1	YOR140W	YES
48	SHO1	YER118C	
49	SKS1	YPL026C	
50	SNF1	YDR477W	YES
51	SPA2	YLL021W	YES
52	SPH1	YLR313C	
53	SSN3	YPL042C	
54	STE11	YLR362W	
55	STE12	YHR084W	
56	STE20	YHL007C	YES
57	STE50	YCL032W	YES
58	STE7	YDL159W	
59	SWE1	YJL187C	
60	TEC1	YBR083W	
61	TMN2	YDR107C	
62	TMN3	YER113C	
63	TPK1	YJL164C	
64	TPK2	YPL203W	
65	TPK3	YKL166C	YES
66	TPM1	YNL079C	YES
67	VPS60	YDR486C	
68	YBR062C	YBR062C	
69	YNL260C	YNL260C	

Table S2. Phosphoproteins having degrees of connectivity greater than 1 in the stringent correlation network.

Index	Standard name	Degree of connectivity	Evidence of involvement in filamentous growth	Remarks	Stress response
1	SEC21	6	-	In the stringent correlation network, SEC1 correlated with KEM1 (known involved in filamentous growth), and ARE2 (indirect evidence of involvement).	*SEC21* overexpression leads to decreased rapamycin resistance. [1]. Sec21p mutants have reduced resistance to tunicamycin decreased [2].
2	ABF1	5	-	In the stringent correlation network, ABF1 correlated with SPT6 (known involved in filamentous growth), and PBP1 (validated by our experiment).	Implicated in oxidative stress [3]. Abf1p mutants exhibit decreased sensitivity to tunicamycin [3].
3	ARE2	5	Are2p has positive physical interactions with Fus3p, Tpk1p and Ste20p [4].	Several other proteins involved in sterol biosynthesis or response are differentially phosphorylated in the kinase-dead mutants (YML008C, YHR073W, YKL140W)	Are2p is linked to cell wall construction and plays a key role in sterol biosynthesis whose regulation is important in specific stress responses [5,6].
4	DCP2	5	(1) Dcp2p has physical interactions with Spt6p [7] and Kem1p [7–9], both are involved in filamentous growth. (2) Dcp2p is a phosphorylation substrate of Ste20p [10].	In the stringent correlation network, DCP2 is highly correlated with SUM1 (positive) and ARE2 (negative).	Dcp2p is involved in stress granule assembly [10].
5	KEM1 (Alias XRN1)	5	Kem1p plays a direct role in yeast filamentous growth, affecting *FLO11* transcription [11].		*KEM1* deletion has increased sensitivity to hyperosmotic stress [12].
6	NUP145	5	-	In the stringent correlation network, NUP145 is highly correlated with SPT6 (known involved in filamentous growth) and HSP42(indirect evidence).	*NUP145* deletion has decreased resistance to sodium arsenite [13]. Reduced functioning of Nup145p causes the strain to also have decreased resistance to tunicamycin [2].
7	SPA2	5	When *SPA2* is disrupted, filamentous growth decreases [14]. Refer to SGD.		Required for recovery from osmotic stress [15].
8	CHO1	4	-		
9	GLY1	4	-	In the stringent correlation network, GLY1 has positive correlation with KEM1 and SPA2, both known	The *GLY1* deletion strain has decreased resistance to hyperosmotic stress [16].

				to be involved in filamentous growth.	
10	HSP42	4	Hsp42p has physical association with Fus3p [17].		Protein expression is induced by stresses such as heat shock, salt shock and starvation [18], which might also contribute to the phosphorylation level change that we detected.
11	PWP1	4	-	In the stringent correlation network, PWP1 has positive correlation with SPT6.	Pwp1p mutants have decreased resistance to tunicamycin [2].
12	PUF6	3	-		The *PUF6* deletion strain has decreased resistance to multiple chemical stresses [19–21] and is more sensitive to both cold [22] and heat [23].
13	SPT6	3	(1) Filamentous growth decreases when *SPT6* is disrupted [14]. Refer to SGD. (2) *SPT6* has a genetic interaction with *RAS2* [24] which regulates filamentous growth [25]. (3) Spt6p has physical interaction with Kss1p [4].		Spt6p is involved in regulation of transcription from RNA polymerase II promoter in response to stress [26].
14	SSD1	3	(1) Ssd1p has physical interactions with Ste20p [4] and Kem1p [27]. (2) *SSD1* has positive genetic interaction with *KEM1* [28], and negative genetic interaction with *STE50* [29], known to be involved in filamentous growth [30]. (3) *SSD1* also has negative genetic interaction with *ILM1* [31]. Ilm1p is required for slowed DNA synthesis-induced filamentous growth [32,33]. (4) Overexpression of the *SSD1* homologue, ropy, in N. crassa has been shown to suppress mutations of POD6 and COT1 which play essential roles in hyphal tip extension [34], a process required for filamentous growth.		Required for thermotolerance [35] and migrates to stress granules [36].
15	SUM1	3	*SUM1* has a negative	In the stringent	Involved in osmotic

			genetic interaction with *ELM1* and *TPK3* [37].	correlation network, SUM1 has positive correlation with SPA2 and KEM1, both known to be involved in filamentous growth.	stress [38]. Predicted to be involved in stress response [39].
16	NUP2	2	-	In the stringent correlation network, NUP2 are positively correlated PBP1(predicted and validated by our experiment).	Nup2p is involved in mRNA export from nucleus in response to heat stress [40]. When Nup2 is deleted, the strain has decreased resistance to bortezomib [41] and arsenite(3-) [42].
17	PBI2	2	Pbi2p has not been reported related to filamentous growth. However, our experiment has validated that *PBI2* mutant strain have decreased filamentous growth under 1% butanol treatment, which will induce filamentous growth in WT haploid strain.		*PBI2* deletion has decreased resistance to hperosmotic stress [16] and multiple chemicals [19,20,43].
18	PBP1	2			Pbp1p is involved in stress granule assembly [44]. *PBP1* mutant has decreased resistance to stress.
19	UFD1	2	*UFD1* has a negative genetic interaction with *KEM1* [45].	In the stringent correlation network, UPD1 has positive correlation with KEM1 and SPA2, both known to be involved in filamentous growth.	Involved in ER and heat stress responses [46,47].

Table S3. Inferred causal relationships.

Index	Inferred relationships	Probability	From protein & peptide	To protein & peptide	Remarks
1	SNF1 => PBP1	0.75949	SNF1 _SVSDELDTFLSQS(ph)PPTFQQQSK_	PBP1 _VADSGVSDS(ph)VDDLAK_	
2	SNF1 => PBP1	0.75949	SNF1 _SVSDELDTFLSQSPPTFQQQS(ph)K_	PBP1 _VADSGVSDS(ph)VDDLAK_	
3	HSP42 => STE20	0.70946	HSP42 _KS(ph)S(ph)SFAHLQAPSPIPDPLQVSKPETR_	STE20 _HQQPVASSTVNS(ph)NK_	
4	HSP42 => STE20	0.70946	HSP42 _KS(ph)SSFAHLQAPSPIPDPLQVSKPETR_	STE20 _HQQPVASSTVNS(ph)NK_	
5	PBP1 => STE20	0.70946	PBP1 _VADSGVSDS(ph)VDDLAK_	STE20 _HQQPVASSTVNS(ph)NK_	
6	SEC21 => STE20	0.70946	SEC21 _SETTLDTT(ph)PEAESVPEKR_	STE20 _HQQPVASSTVNS(ph)NK_	
7	ARE2 => STE20	0.70946	ARE2 _KSS(ph)PDAVDSVGK_	STE20 _HQQPVASSTVNS(ph)NK_	Protein physical interaction [4]
8	PDR12 => STE20	0.70946	PDR12 _KEMDS(ph)FEINDLDFDLR_	STE20 _HQQPVASSTVNS(ph)NK_	
9	STE20 => SSD1	0.66372	STE20 _HQQPVASSTVNS(ph)NK_	SSD1 _NQSQQPQQQLS(ph)PFR_	Protein physical interaction [4]
10	SNF1 => TPM1	0.58252	SNF1 _SVSDELDTFLSQS(ph)PPTFQQQSK_	TPM1 _KELDEIAAS(ph)LENL_	
11	SNF1 => RCN2	0.58252	SNF1 _SVSDELDTFLSQS(ph)PPTFQQQSK_	RCN2 _SSQTS(ph)LPSQLENK_	
12	SNF1 => RCN2	0.58252	SNF1 _SVSDELDTFLSQS(ph)PPTFQQQSK_	RCN2 _SSQTSLPS(ph)QLENKDK_	
13	SNF1 => TPM1	0.58252	SNF1 _SVSDELDTFLSQSPPTFQQQS(ph)K_	TPM1 _KELDEIAAS(ph)LENL_	
14	SNF1 => RCN2	0.58252	SNF1 _SVSDELDTFLSQSPPTFQQQS(ph)K_	RCN2 _SSQTS(ph)LPSQLENK_	
15	SNF1 => RCN2	0.58252	SNF1 _SVSDELDTFLSQSPPTFQQQS(ph)K_	RCN2 _SSQTSLPS(ph)QLENKDK_	
16	STE20 => PDR12	0.58252	STE20 _RAT(ph)PVS(ph)TPVISKPSMTTTPR_	PDR12 _HLSNILS(ph)NEEGIER_	
17	STE20 => NUP145	0.58252	STE20 _RAT(ph)PVS(ph)TPVISKPSMTTTPR_	NUP145 _AYEPDLSDADFEGIEAS(ph)PK_	
18	STE20 => PBP1	0.58252	STE20 _RAT(ph)PVS(ph)TPVISKPSMTTTPR_	PBP1 _S(ph)GSNISQGQSSTGHTTR_	
19	STE50 => SNF1	0.54404	STE50 _RES(ph)PVTVFR_	SNF1 _SVSDELDTFLSQS(ph)PPTFQQQSK _	Negative genetics interaction [48]
20	STE50 => SNF1	0.54404	STE50 _RES(ph)PVTVFR_	SNF1 _SVSDELDTFLSQSPPTFQQQS(ph)K _	
21	HSP42 => STE20	0.54404	HSP42 _KS(ph)S(ph)SFAHLQAPSPIPDPLQVSKPETR_	STE20 _RAT(ph)PVS(ph)TPVISKPSMTTTPR _	
22	SSD1 => SNF1	0.54404	SSD1 _SST(ph)INNDSDSLSSPTK_	SNF1 _SVSDELDTFLSQS(ph)PPTFQQQSK	Protein physical interaction [4]

				_	
23	SSD1 => SNF1	0.54404	SSD1 _SST(ph)INNDSDSLSSPTK_	SNF1 _SVSDELDTFLSQSPPTFQQQS(ph)K_	
24	SUM1 => SNF1	0.54404	SUM1 _TAGDDGALDQTENTSIS(ph)PK_	SNF1 _SVSDELDTFLSQS(ph)PPTFQQQSK_	
25	SUM1 => SNF1	0.54404	SUM1 _TAGDDGALDQTENTSIS(ph)PK_	SNF1 _SVSDELDTFLSQSPPTFQQQS(ph)K_	
26	SUM1 => STE20	0.54404	SUM1 _TAGDDGALDQTENTSIS(ph)PK_	STE20 _RAT(ph)PVS(ph)TPVISKPSMTTTPR_	
27	CHO1 => STE20	0.54404	CHO1 _DENDGYAS(ph)DEVGGTLSR_	STE20 _RAT(ph)PVS(ph)TPVISKPSMTTTPR_	
28	NUP145 => STE20	0.54404	NUP145 _TDGTFGTLSGKDDS(ph)IVEEK_	STE20 _RAT(ph)PVS(ph)TPVISKPSMTTTPR_	
29	PBP1 => STE20	0.54404	PBP1 _VADSGVSDS(ph)VDDLAK_	STE20 _RAT(ph)PVS(ph)TPVISKPSMTTTPR_	
30	CHO1 => SNF1	0.54404	CHO1 _DENDGYAS(ph)DEVGGTLSR_	SNF1 _SVSDELDTFLSQS(ph)PPTFQQQSK_	
31	NUP145 => SNF1	0.54404	NUP145 _TDGTFGTLSGKDDS(ph)IVEEK_	SNF1 _SVSDELDTFLSQS(ph)PPTFQQQSK_	
32	PWP1 => SNF1	0.54404	PWP1 _ATLEEAEGES(ph)GVEDDAATGSSNK_	SNF1 _SVSDELDTFLSQS(ph)PPTFQQQSK_	
33	RAS2 => SNF1	0.54404	RAS2 _NVNSS(ph)TTVVNAR_	SNF1 _SVSDELDTFLSQS(ph)PPTFQQQSK_	Negative genetic interaction [48]
34	DCP2 => SNF1	0.54404	DCP2 _NPISSTVSSNQQS(ph)PK_	SNF1 _SVSDELDTFLSQS(ph)PPTFQQQSK_	
35	RCN2 => SNF1	0.54404	RCN2 _NKPLLSINT(ph)DPGVTGVDSSSLNK_	SNF1 _SVSDELDTFLSQS(ph)PPTFQQQSK_	
36	CHO1 => SNF1	0.54404	CHO1 _DENDGYAS(ph)DEVGGTLSR_	SNF1 _SVSDELDTFLSQSPPTFQQQS(ph)K	

				_	
37	NUP145 => SNF1	0.54404	NUP145 _TDGTFGTLSGKDDS(ph)IVEEK_	SNF1 _SVSDELDTFLSQSPPTFQQQS(ph)K_	
38	PWP1 => SNF1	0.54404	PWP1 _ATLEEAEGES(ph)GVEDDAATGSSNK_	SNF1 _SVSDELDTFLSQSPPTFQQQS(ph)K_	
39	RAS2 => SNF1	0.54404	RAS2 _NVNSS(ph)TTVVNAR_	SNF1 _SVSDELDTFLSQSPPTFQQQS(ph)K_	Negative genetic interaction [48]
40	DCP2 => SNF1	0.54404	DCP2 _NPISSTVSSNQQS(ph)PK_	SNF1 _SVSDELDTFLSQSPPTFQQQS(ph)K_	
41	RCN2 => SNF1	0.54404	RCN2 _NKPLLSINT(ph)DPGVTGVDSSSLNK_	SNF1 _SVSDELDTFLSQSPPTFQQQS(ph)K_	Negative genetic interaction [48]
42	PWP1 => STE20	0.54404	PWP1 _ATLEEAEGES(ph)GVEDDAATGSSNK_	STE20 _RAT(ph)PVS(ph)TPVISKPSMTTTPR_	
43	RCN2 => STE20	0.54404	RCN2 _NKPLLSINT(ph)DPGVTGVDSSSLNK_	STE20 _RAT(ph)PVS(ph)TPVISKPSMTTTPR_	Rcn2p and Bmh1p have physical interaction captured by affinity capture-MS [17], while Bmh1p associates with Ste20p to influence filamentous growth [49].
44	PBP1 => STE20	0.51655	PBP1 _S(ph)GSNISQGQSSTGHTTR_	STE20 _LSLTDS(ph)TETIENNATVK_	

Table S4. Dataset constitution.

	**Samples**
**Three replicates**	WT/SNF1-KD and WT/TPK2-KD
**Two replicates**	WT/ELM1-KD, WT/FUS3-KD, WT/SKS1-KD, WT/STE20-KD and WT/KSP1-KD
**No replicates**	WT/KSS1-KD

Mutants and WTs were mixed for triplex SILAC experiments.

Supplementary Figures

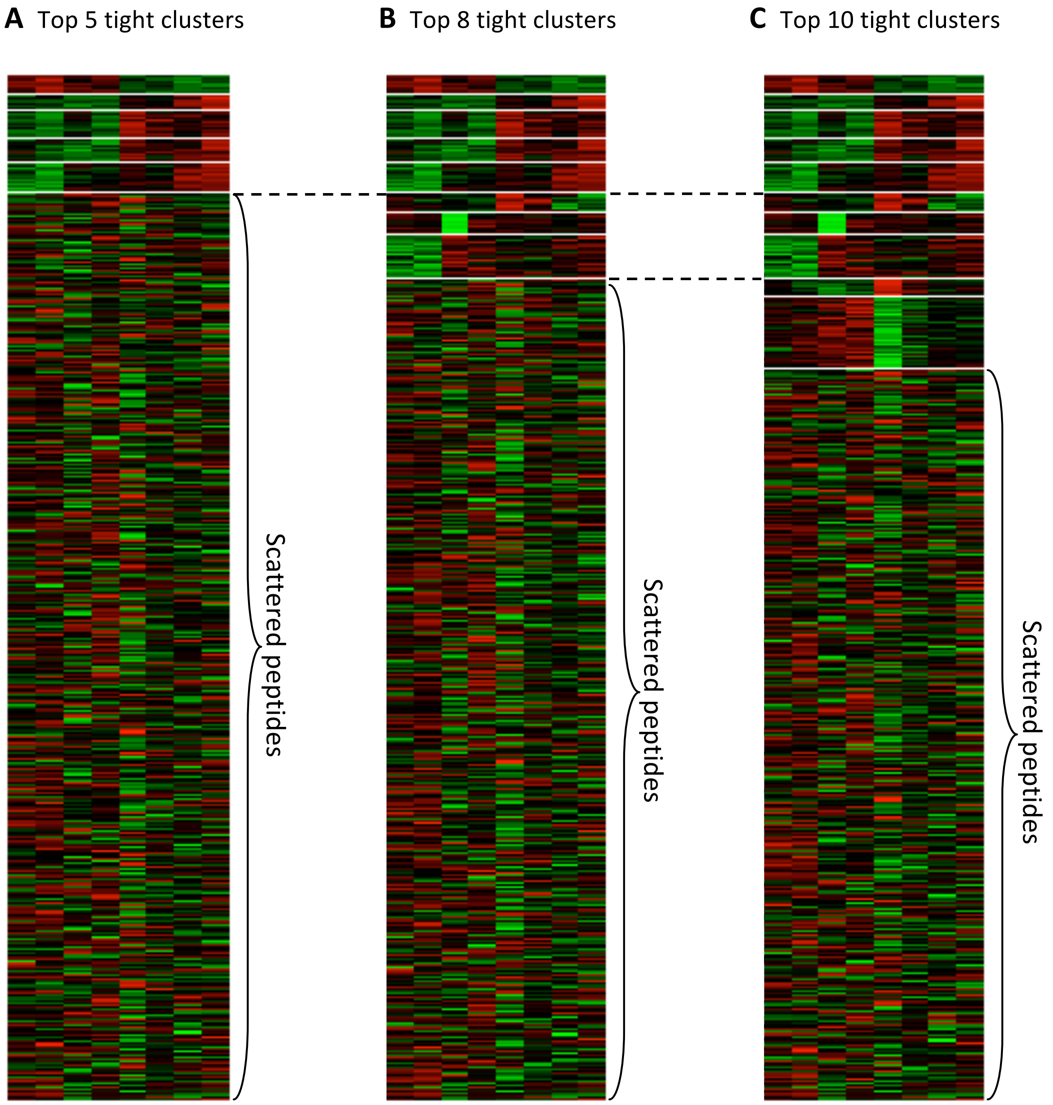

Figure S1. Top clusters selected by tight clustering.
The phosphopeptides commonly identified in 4 – 8 KD-versus-WT conditions were used. After missing value being imputed, the tight cluster method [50] was used to pick out the top tightest and stablest clusters. R package tightClust was used, adopting the suggested parameters. R code: tight.clust(data.impute, target, k.min=15, random.seed=12345), the value of target is 5, 8 and 10, respectively, for **A**, **B** and **C**. The columns of a map correspond to SKS1-KD, STE20-KD, SNF1-KD, TPK2-KD, ELM1-KD, FUS3-KD, KSS1-KD and KSP1-KD. The rows correspond to phosphopeptides. **A** - Top 5 tight clusters were sequentially selected, and plotted on the top. The order of stability decreases from the top down. Scattered peptides were not clustered. **B** - Top 8 tight clusters sequentially selected. **C** - Top 10 tight clusters sequentially selected.

A Confidence view

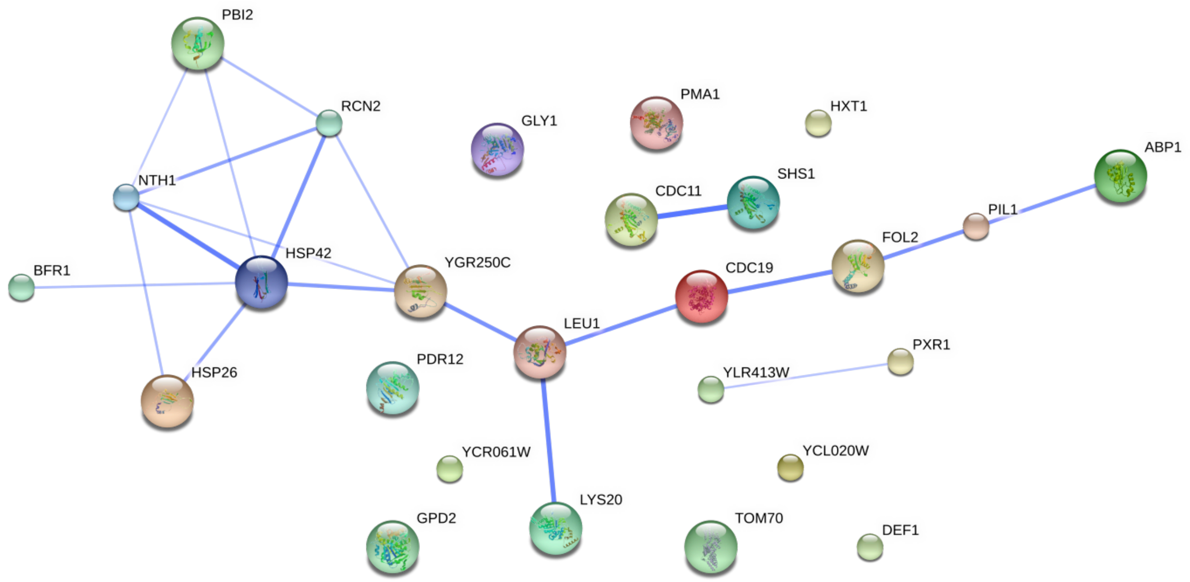

B Evidence view

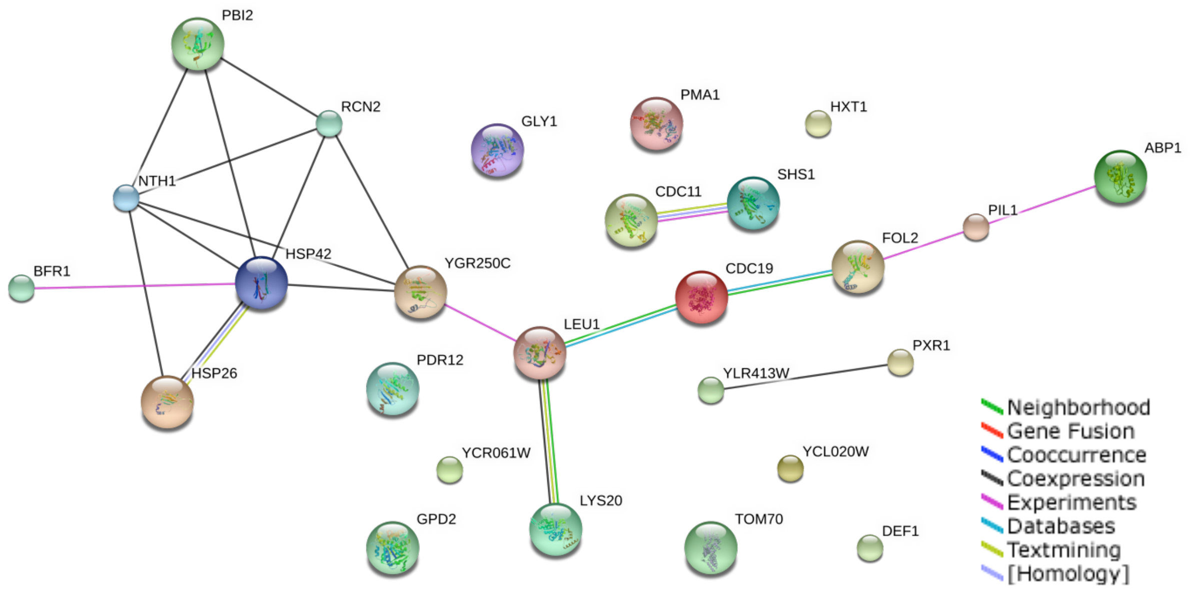

Figure S2. STRING reported inner connections of the globally significant proteins.
The network was generated using STRING v9.0 [51,52] using the default parameters with median confidence. **A** – Confidence view. The thicker the edge it, the more confidence the interaction is. **B** – Evidence view. Different colors of the edges indicate different evidence types.

References

1. Butcher RA, Bhullar BS, Perlstein EO, Marsischky G, LaBaer J, et al. (2006) Microarray-based method for monitoring yeast overexpression strains reveals small-molecule targets in TOR pathway. Nature chemical biology 2: 103–109. doi:10.1038/nchembio762.

2. Breslow DK, Cameron DM, Collins SR, Schuldiner M, Stewart-Ornstein J, et al. (2008) A comprehensive strategy enabling high-resolution functional analysis of the yeast genome. Nature methods 5: 711–718. doi:10.1038/nmeth.1234.

3. Woo DK, Phang TL, Trawick JD, Poyton RO (2009) Multiple pathways of mitochondrial-nuclear communication in yeast: intergenomic signaling involves ABF1 and affects a different set of genes than retrograde regulation. Biochimica et Biophysica Acta 1789: 135–145.

4. Ptacek J, Devgan G, Michaud G, Zhu H, Zhu X, et al. (2005) Global analysis of protein phosphorylation in yeast. Nature 438: 679–684. doi:10.1038/nature04187.

5. Borah S, Shivarathri R, Kaur R (2011) The Rho1 GTPase-activating protein CgBem2 is required for survival of azole stress in Candida glabrata. The Journal of biological chemistry 286: 34311–34324. doi:10.1074/jbc.M111.264671.

6. Montañés FM, Pascual-Ahuir A, Proft M (2011) Repression of ergosterol biosynthesis is essential for stress resistance and is mediated by the Hog1 MAP kinase and the Mot3 and Rox1 transcription factors. Molecular Microbiology 79: 1008–1023. doi:10.1111/j.1365-2958.2010.07502.x.

7. Fromont-Racine M, Mayes AE, Brunet-Simon A, Rain JC, Colley A, et al. (2000) Genome-wide protein interaction screens reveal functional networks involving Sm-like proteins. Yeast (Chichester, England) 17: 95–110. doi:10.1002/1097-0061(20000630)17:2<95::AID-YEA16>3.0.CO;2-H.

8. Gavin A-C, Aloy P, Grandi P, Krause R, Boesche M, et al. (2006) Proteome survey reveals modularity of the yeast cell machinery. Nature 440: 631–636. doi:10.1038/nature04532.

9. Collins SR, Kemmeren P, Zhao X-C, Greenblatt JF, Spencer F, et al. (2007) Toward a comprehensive atlas of the physical interactome of Saccharomyces cerevisiae. Molecular & cellular proteomics : MCP 6: 439–450. doi:10.1074/mcp.M600381-MCP200.

10. Yoon J-H, Choi E-J, Parker R (2010) Dcp2 phosphorylation by Ste20 modulates stress granule assembly and mRNA decay in Saccharomyces cerevisiae. The Journal of cell biology 189: 813–827. doi:10.1083/jcb.200912019.

11. Kim J, Kim J (2002) KEM1 is involved in filamentous growth of Saccharomyces cerevisiae. FEMS microbiology letters 216: 33–38.

12. Cai H, Kauffman S, Naider F, Becker JM (2006) Genomewide screen reveals a wide regulatory network for di/tripeptide utilization in Saccharomyces cerevisiae. Genetics 172: 1459–1476. doi:10.1534/genetics.105.053041.

13. Pan X, Reissman S, Douglas NR, Huang Z, Yuan DS, et al. (2010) Trivalent arsenic inhibits the functions of chaperonin complex. Genetics 186: 725–734. doi:10.1534/genetics.110.117655.

14. Jin R, Dobry CJ, McCown PJ, Kumar A (2008) Large-scale analysis of yeast filamentous growth by systematic gene disruption and overexpression. Molecular biology of the cell 19: 284–296. doi:10.1091/mbc.E07-05-0519.

15. Yuzyuk T, Amberg DC (2003) Actin Recovery and Bud Emergence in Osmotically Stressed Cells Requires the Conserved Actin Interacting Mitogen-activated Protein Kinase Kinase Kinase Ssk2p/MTK1 and the Scaffold Protein Spa2p. Molecular Biology of the Cell 14: 3013–3026.

16. Yoshikawa K, Tanaka T, Furusawa C, Nagahisa K, Hirasawa T, et al. (2009) Comprehensive phenotypic analysis for identification of genes affecting growth under ethanol stress in Saccharomyces cerevisiae. FEMS yeast research 9: 32–44. doi:10.1111/j.1567-1364.2008.00456.x.

17. Krogan NJ, Cagney G, Yu H, Zhong G, Guo X, et al. (2006) Global landscape of protein complexes in the yeast Saccharomyces cerevisiae. Nature 440: 637–643. doi:10.1038/nature04670.

18. Wotton D, Freeman K, Shore D (1996) Multimerization of Hsp42p, a novel heat shock protein of Saccharomyces cerevisiae, is dependent on a conserved carboxyl-terminal sequence. The Journal of biological chemistry 271: 2717–2723.

19. Cherry JM, Hong EL, Amundsen C, Balakrishnan R, Binkley G, et al. (2011) Saccharomyces Genome Database: the genomics resource of budding yeast. Nucleic acids research. doi:10.1093/nar/gkr1029.

20. Parsons AB, Brost RL, Ding H, Li Z, Zhang C, et al. (2004) Integration of chemical-genetic and genetic interaction data links bioactive compounds to cellular target pathways. Nature biotechnology 22: 62–69. doi:10.1038/nbt919.

21. Kapitzky L, Beltrao P, Berens TJ, Gassner N, Zhou C, et al. (2010) Cross-species chemogenomic profiling reveals evolutionarily conserved drug mode of action. Molecular systems biology 6: 451.

22. Li Z, Lee I, Moradi E, Hung N-J, Johnson AW, et al. (2009) Rational extension of the ribosome biogenesis pathway using network-guided genetics. PLoS biology 7: e1000213.

23. Sinha H, David L, Pascon RC, Clauder-Münster S, Krishnakumar S, et al. (2008) Sequential elimination of major-effect contributors identifies additional quantitative trait loci conditioning high-temperature growth in yeast. Genetics 180: 1661–1670.

24. Howard SC, Hester A, Herman PK (2003) The Ras/PKA signaling pathway may control RNA polymerase II elongation via the Spt4p/Spt5p complex in Saccharomyces cerevisiae. Genetics 165: 1059–1070.

25. Gimeno CJ, Ljungdahl PO, Styles CA, Fink GR (1992) Unipolar cell divisions in the yeast S. cerevisiae lead to filamentous growth: regulation by starvation and RAS. Cell 68: 1077–1090.

26. Klopf E, Paskova L, Solé C, Mas G, Petryshyn A, et al. (2009) Cooperation between the INO80 complex and histone chaperones determines adaptation of stress gene transcription in the yeast

Saccharomyces cerevisiae. Molecular and cellular biology 29: 4994–5007. doi:10.1128/MCB.01858-08.

27. Kurischko C, Kim HK, Kuravi VK, Pratzka J, Luca FC (2011) The yeast Cbk1 kinase regulates mRNA localization via the mRNA-binding protein Ssd1. The Journal of cell biology 192: 583–598. doi:10.1083/jcb.201011061.

28. Koh JLY, Ding H, Costanzo M, Baryshnikova A, Toufighi K, et al. (2010) DRYGIN: a database of quantitative genetic interaction networks in yeast. Nucleic acids research 38: D502–7. doi:10.1093/nar/gkp820.

29. Fiedler D, Braberg H, Mehta M, Chechik G, Cagney G, et al. (2009) Functional organization of the S. cerevisiae phosphorylation network. Cell 136: 952–963. doi:10.1016/j.cell.2008.12.039.

30. Ramezani Rad M, Jansen G, Bühring F, Hollenberg CP (1998) Ste50p is involved in regulating filamentous growth in the yeast Saccharomyces cerevisiae and associates with Ste11p. Molecular & general genetics : MGG 259: 29–38.

31. Hoppins S, Collins SR, Cassidy-Stone A, Hummel E, DeVay RM, et al. (2011) A mitochondrial-focused genetic interaction map reveals a scaffold-like complex required for inner membrane organization in mitochondria. The Journal of Cell Biology 195: 323–340. doi:10.1083/jcb.201107053.

32. Entian KD, Schuster T, Hegemann JH, Becher D, Feldmann H, et al. (1999) Functional analysis of 150 deletion mutants in Saccharomyces cerevisiae by a systematic approach. Molecular & general genetics : MGG 262: 683–702.

33. Kang CM, Jiang YW (2005) Genome-wide survey of non-essential genes required for slowed DNA synthesis-induced filamentous growth in yeast. Yeast (Chichester, England) 22: 79–90. doi:10.1002/yea.1195.

34. Seiler S, Vogt N, Ziv C, Gorovits R, Yarden O (2006) The STE20/germinal center kinase POD6 interacts with the NDR kinase COT1 and is involved in polar tip extension in Neurospora crassa. Molecular Biology of the Cell 17: 4080–4092.

35. Mir SS, Fiedler D, Cashikar AG (2009) Ssd1 Is Required for Thermotolerance and Hsp104-Mediated Protein Disaggregation in Saccharomyces cerevisiae. Molecular and Cellular Biology 29: 187–200.

36. Kurischko C, Kuravi VK, Herbert CJ, Luca FC (2011) Nucleocytoplasmic shuttling of Ssd1 defines the destiny of its bound mRNAs. Molecular Microbiology 81: 831–849. doi:10.1111/j.1365-2958.2011.07731.x.

37. Bandyopadhyay S, Mehta M, Kuo D, Sung M-K, Chuang R, et al. (2010) Rewiring of genetic networks in response to DNA damage. Science (New York, NY) 330: 1385–1389. doi:10.1126/science.1195618.

38. Humphrey T, Enoch T (1998) Sum1, a highly conserved WD-repeat protein, suppresses S-M checkpoint mutants and inhibits the osmotic stress cell cycle response in fission yeast. Genetics 148: 1731–1742.

39. Segal E, Shapira M, Regev A, Pe'er D, Botstein D, et al. (2003) Module networks: identifying regulatory modules and their condition-specific regulators from gene expression data. Nature genetics 34: 166–176. doi:10.1038/ng1165.

40. Thomsen R, Saguez C, Nasser T, Jensen TH (2008) General, rapid, and transcription-dependent fragmentation of nucleolar antigens in S. cerevisiae mRNA export mutants. RNA (New York, NY) 14: 706–716. doi:10.1261/rna.718708.

41. Fleming JA, Lightcap ES, Sadis S, Thoroddsen V, Bulawa CE, et al. (2002) Complementary whole-genome technologies reveal the cellular response to proteasome inhibition by PS-341. Proceedings of the National Academy of Sciences of the United States of America 99: 1461–1466. doi:10.1073/pnas.032516399.

42. Zhou X, Arita A, Ellen TP, Liu X, Bai J, et al. (2009) A genome-wide screen in Saccharomyces cerevisiae reveals pathways affected by arsenic toxicity. Genomics 94: 294–307. doi:10.1016/j.ygeno.2009.07.003.

43. Shin C-S, Kim SY, Huh W-K (2009) TORC1 controls degradation of the transcription factor Stp1, a key effector of the SPS amino-acid-sensing pathway in Saccharomyces cerevisiae. Journal of cell science 122: 2089–2099.

44. Buchan JR, Muhlrad D, Parker R (2008) P bodies promote stress granule assembly in Saccharomyces cerevisiae. The Journal of cell biology 183: 441–455.

45. Costanzo M, Baryshnikova A, Bellay J, Kim Y, Spear ED, et al. (2010) The genetic landscape of a cell. Science (New York, NY) 327: 425–431. doi:10.1126/science.1180823.

46. Chen M, Gutierrez GJ, Ronai ZA (2011) Ubiquitin-recognition protein Ufd1 couples the endoplasmic reticulum (ER) stress response to cell cycle control. Proceedings of the National Academy of Sciences of the United States of America 108: 9119–9124. doi:10.1073/pnas.1100028108.

47. Hsieh M-T, Chen R-H (2011) Cdc48 and Cofactors Npl4-Ufd1 Are Important for G1 Progression during Heat Stress by Maintaining Cell Wall Integrity in Saccharomyces cerevisiae. PLoS ONE 6: 13.

48. Sharifpoor S, Van Dyk D, Costanzo M, Baryshnikova A, Friesen H, et al. (2012) Functional wiring of the yeast kinome revealed by global analysis of genetic network motifs. Genome research 22: 791–801.

49. Roberts RL, Mösch HU, Fink GR (1997) 14-3-3 proteins are essential for RAS/MAPK cascade signaling during pseudohyphal development in S. cerevisiae. Cell 89: 1055–1065.

50. Tseng GC, Wong WH (2005) Tight clustering: a resampling-based approach for identifying stable and tight patterns in data. Biometrics 61: 10–16.

51. Von Mering C, Huynen M, Jaeggi D, Schmidt S, Bork P, et al. (2003) STRING: a database of predicted functional associations between proteins. Nucleic Acids Research 31: 258–261.

52. Szklarczyk D, Franceschini A, Kuhn M, Simonovic M, Roth A, et al. (2011) The STRING database in 2011: functional interaction networks of proteins, globally integrated and scored. Nucleic Acids Research 39: D561–8. doi:10.1093/nar/gkq973.
